# The glycosaminoglycan chains of perlecan regulate the perivascular fluid transport

**DOI:** 10.1186/s12987-025-00648-7

**Published:** 2025-05-08

**Authors:** Abhishek Singh, Mika Kaakinen, Harri Elamaa, Vesa Kiviniemi, Lauri Eklund

**Affiliations:** 1 Oulu Center for Cell-Matrix Research, Faculty of Biochemistry and Molecular Medicine, Biocenter Oulu, University of Oulu, Oulu, Finland; 2 Research Unit of Health Sciences and Technology (HST), Faculty of Medicine, Biocenter Oulu, University of Oulu, Oulu, Finland; 3 Oulu Functional Neuroimaging (OFNI), Diagnostic Imaging, Medical Research Center (MRC), Oulu University Hospital, Oulu, Finland

**Keywords:** Amyloid beta, Basement membrane, Cerebrospinal fluid, Extracellular matrix, Heparan sulphate proteoglycan, Perivascular space, Perlecan, Glycosaminoglycans

## Abstract

**Background:**

The perivascular conduct pathway that connects the cerebrospinal fluid spaces with the interstitial fluid in the parenchyma are of importance for solute clearance from the brain. In this pathway, the relatively wide perivascular space (PVS) surrounding the pial arteries provides a low-resistant passage while around the perforating arteries, the solute movement is along the basement membrane (BM), that prevents the free exchange of interstitial fluids and solutes. We hypothesize that this selectivity involves specific components of the vascular BM, which is mainly composed of type IV collagen (Col IV) and laminin networks interconnected by nidogens and heparan sulphate proteoglycans (HSPGs). Perlecan is the major HSPG in the BM that binds to Col IV and laminin via glycosaminoglycan (GAG) chains to form a molecular sieve. GAGs may also provide the charge selectivity required for filtration, and also a scaffold for amyloid-β (Aβ) aggregation. The purpose of this study was the functional characterization of perivascular fluid transport and brain clearance in mice lacking perlecan GAG chains.

**Methods:**

We generated a novel mouse line (*Hspg2*^∆3∆91^) lacking perlecan GAG side chains and investigated perivascular flow and brain clearance in these mice using intravital multiphoton and fluorescence recovery after photobleaching techniques, and functional assays with various tracers. Potentially deleterious effects on brain homeostasis were investigated using transcriptomic, proteomic and immunohistochemical methods. The *Hspg2*^∆3∆91^ mice were crossed with a *5xFAD* line to examine the importance of GAGs in Aβ aggregation.

**Results:**

We observed a delayed inflow of CSF tracer into the *Hspg2*^∆3∆91^ brain with no changes in the clearance of parenchymal injected tracers. Quantification of the Aβ plaques revealed fewer and smaller plaques in the walls of the pial arteries at six months of age, but not in the brain parenchyma. Surprisingly, perlecan GAG deficiency had no severe deleterious effects on brain homeostasis in transcriptomic and proteomic analyses.

**Conclusions:**

Potential brain clearance mechanisms are dependent on the flow through special ECM structures. BM is mainly known for its barrier function, whereas very little is known about how passage along the perivascular ECM is established. This study shows that the GAG composition of the BM affects the solute dynamics and Aβ deposition in the periarterial space.

**Supplementary Information:**

The online version contains supplementary material available at 10.1186/s12987-025-00648-7.

## Background

The central nervous system (CNS) is surrounded by cerebrospinal fluid (CSF), which protects the brain from injuries, provides nutrients and functions as a reservoir for metabolic waste removal from the brain parenchyma. Previous multiphoton and electron microscopy studies have identified the perivascular space (PVS) as a conductive pathway between the CSF and the parenchymal neuronal tissues along which solutes can be distributed within the CNS [1, 2]. The PVS on the brain surface can be defined as a relatively low-resistant pathway [3], characterized by the presence of fibrillar collagen types I and III and the reticular (pial) fibroblast marker, ERTR7, expressing cells [4, 5], while the PVS around the penetrating arteries/arterioles, at the site where the pial cell-defined PVS terminates [4, 5], is composed of a basement membrane (BM) that regulates the dynamics of solute movement in a size and charge-dependent manner [2, 4]. While it remains unclear whether there is a fluid space existing around the arteriole and peri-capillary compartments, the presence of CSF-injected tracers around these vessels [4, 6] suggests a continuation of the fluid pathway from the CSF space.

Brain vascular BM is produced by endothelial, smooth muscle and glial cells (astrocytes), the latter bordering on the parenchymal side of the PVS. Interestingly, the solutes in the brain parenchyma and in the CSF appear to follow different pathways along the BMs [7]. Accordingly, tracers injected into the brain parenchyma are found within the BM surrounding the capillaries and arterial smooth muscle cells while those injected into the CSF additionally occupy the outer aspects of the PVS, the pial-glial BM [1, 2, 4, 7, 8]. The molecular composition of the BM varies across the PVS [4, 8], although it remains unclear whether these differences influence solute transport. The major constituents of the BMs are type IV collagen (Col IV), laminins, nidogens, and heparan sulphate proteoglycans (HSPGs). Of the three main BM HSPGs (agrin, Col XVIII and perlecan), it is perlecan (HSPG2) that is most abundant [9], consisting of large, complex core proteins and N and C-terminal attachment sites for glycosaminoglycan (GAG) chains [10]. The putative functions mediated by the GAGs that are of importance for the selective passage of solutes between the tissue compartments include collateral interactions among the BM components to form a three-dimensional network that provides them with a poroelastic nature [11]. GAG chains may also be largely responsible for hydration of the BM [11], and significantly impact the diffusivity of the charged particles [12]. Apart from their structural role, perlecan GAGs may also be of functional importance in binding and presenting growth factors for their cell membrane receptors, as is necessary for the development of the perivascular environment for CSF transport [13–15]. In addition to their physiological roles, HSPGs have been implicated in the pathogenesis of Alzheimer’s disease (AD), as they have been shown to accumulate in senile plaques [16–18]. Interestingly, the expression of components of BM, including perlecan, has been reported to be significantly altered in human brains with AD pathology [19–26], implying a possible role of BM in AD pathogenesis. Supporting this, in vitro experiments have demonstrated that perlecan GAG chains can stabilize Aβ fibril formation [27], although the in vivo importance of this is not currently known.

As stated above, previous studies suggest that GAG chains may contribute to solute movement within the BM of the cerebral vasculature and may affect amyloid pathogenesis. To investigate this hypothesis, we generated a mouse line deficient in GAG binding sites on exons 3 and 91 (*Hspg2*^Δ3Δ91^), resulting in the complete absence of GAG chains from the perlecan protein. We then used these *Hspg2*^Δ3Δ91^ mice to investigate the effect of perlecan GAG deficiency on perivascular solute movement and brain clearance. In addition, we crossed the *Hspg2*^∆3∆91^ mice with a *5xFAD* line to examine the importance of perlecan GAG chains in Aβ aggregation.

## Results

### Generation and characterization of a mouse line lacking in glycosaminoglycan chains from the Perlecan core protein

**Fig. 1:**
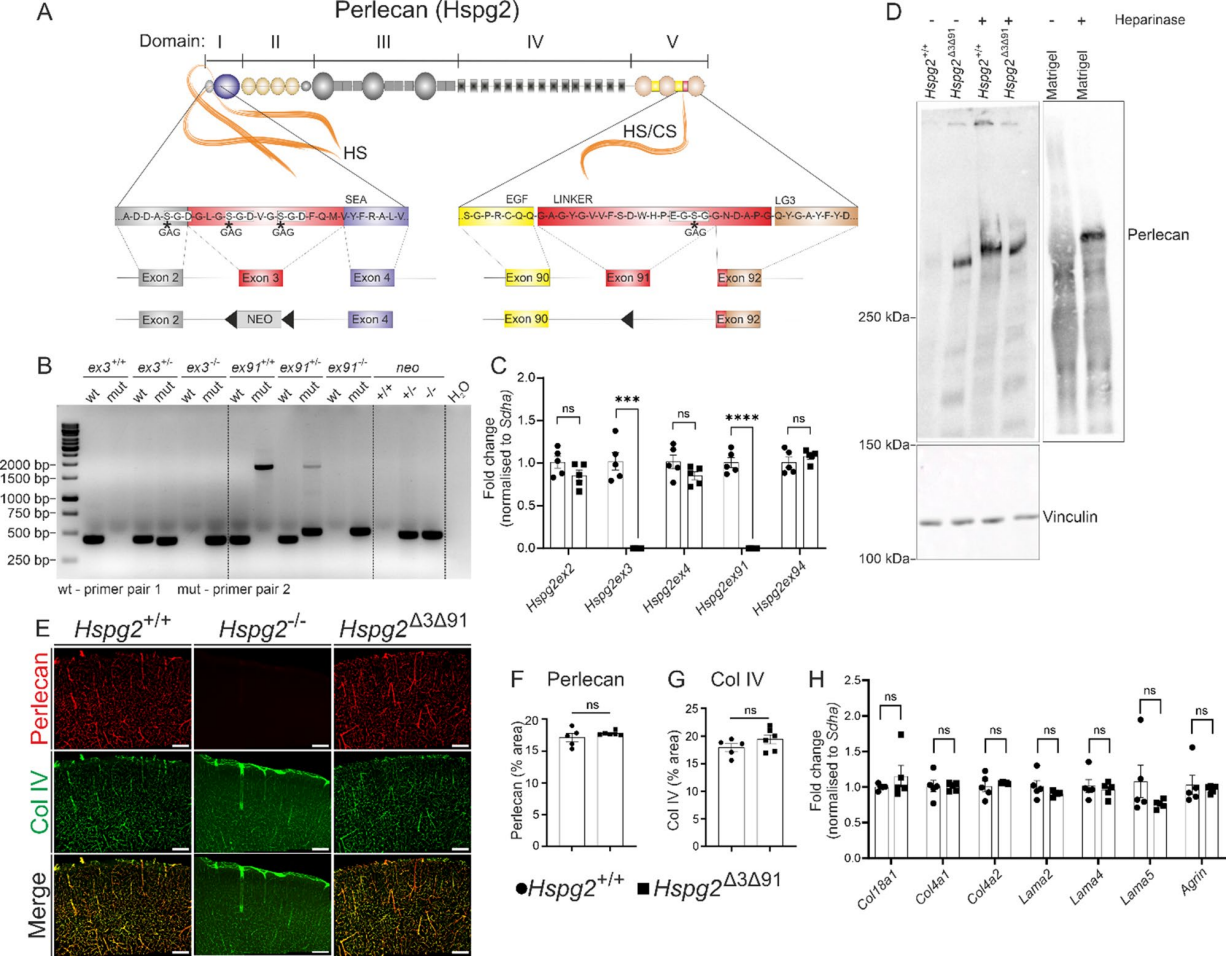
**Generation and characterization of mice lacking glycosaminoglycan chains from perlecan core protein. A**) Perlecan primary structure. Serine residues (S, asterisks) in domains I and V are predicted attachment sites for glycosaminoglycan (GAG) chains. Exons 3 and 91 (red boxes) were removed from the perlecan gene (Hspg2) and exon 3 was replaced with a neo cassette (grey box). Black triangles, loxP sequences. **B**) Genotyping of Hspg2^+/+^, Hspg2^+/-^ and Hspg2^∆3∆91^ mice. **C**) Primers from the targeted exons 3 and 91 showed no qPCR amplicon in the Hspg2^∆3∆91^ mice, indicating the absence of GAG attachment sites. qPCR using primers from exons 2, 4 or 94 showed no differences between the genotypes, demonstrating unaltered Hspg2 mRNA expression (*n* = 5 mice per genotype). **D**) Brain homogenates were treated with heparinase (+) or left untreated (-) and analysed by western blot using anti-perlecan antibody. In the control samples (Hspg2^+/+^ and Matrigel^®^) perlecan is visible in western blot only after removal of the GAGs (heparinase +). Perlecan core protein is readily detectable in the Hspg2^∆3∆91^ mice without heparinase, and the treatment does not affect perlecan mobility, indicating the absence of GAGs in the Hspg2^∆3∆91^ protein. Vinculin was used as a loading control. **E**) Immunofluorescence staining of perlecan core protein (red) and Col IV (green). Perlecan is located in the perivascular BMs of the Hspg2^+/+^ and Hspg2^∆3∆91^ mice, quantification of the data in **F** and **G** (*n* = 5–6 per genotype). There is no perlecan immunoreactivity in the mice devoid of perlecan core protein (Hspg2^-/-^). H) qPCR of the brain hemisphere shows no differences in the expression level of basement membrane components in Hspg2^∆3∆91^ (*n* = 5) mice relative to Hspg2^+/+^ (*n* = 5). Scale bar, 200 μm (**E**). The statistical tests used were the multiple unpaired two-tailed t-test with Welch’s correction followed by Benjamini and Hochberg correction for multiple tests (**C**, **H**), the Mann-Whitney unpaired two-tailed t-test (**F**) and the unpaired two-tailed t-test with Welch’s correction (**G**). ***p < 0.001, ****p < 0.0001. ns, non-significant. Mean ± SEM

Perlecan core protein deficiency is lethal in mice as it leads to cardiac malformations and severe chondrodysplasia [28–30]. As GAG chains are functional moieties that may contribute to the selective passage of solutes through the BM and affect the formation of perivascular ECM [11, 12, 14], we were interested in generating a mouse line to address the specific importance of perlecan GAG side chains for perivascular transport. As shown in Fig. 1A, perlecan protein consists of 5 domains, among which domain I (Ser-Gly-Asp, SGD) and domain V (Glu-Gly-Ser-Gly, EGSG) have sites for the linkage of GAGs at the serine residue [10, 31, 32]. Using gene targeting in embryonic mouse stem cells, we removed the SGD triplet encoding exon 3 (ex3) and the EGSG encoding exon 91 (ex91) from the *Hspg2* gene, thereby generating a mouse model lacking in GAG chain attachment sites (*Hspg2*^∆3∆91^). The SGD triplet, split between ex2 and ex3, is also expected to be disrupted (Fig. 1A), thus achieving removal of all the putative GAG attachment sites from the perlecan core protein. PCR primer pairs were designed to anneal to wild-type (wt) and mutated (mut) exons for mouse genotyping (Fig. 1B). Analysis of homozygous *Hspg2*^∆3∆91^ mice indicated that, in contrast to the perlecan core protein deficient mice, deletion of the GAG attachment sites did not lead to lethality, as the homozygous *Hspg2*^∆3∆91^ mice were born in the expected Mendelian ratio (1:2:1) and without any other readily apparent phenotypic change. Likewise, deletion of the attachment sites did not induce changes related to the expression of any other BM component in the brain (Fig. 1E-H). Moreover, we similarly did not observe any significant alterations in the transcriptomic or proteomic profile of the brain in *Hspg2*^∆3∆91^ mice (Supplementary Fig. 1). Primer pairs designed to multiply mRNA sequences corresponding to the deleted exons ex3 and ex91 showed that these sequences are not included in *Hspg2*^∆3∆91^ mRNA (Fig. 1C), indicating successful removal of GAG binding sites from the *Hspg2*^∆3∆91^ gene. To confirm the GAG deficiency, proteins isolated from the brain hemispheres of *Hspg2*^+/+^ and *Hspg2*^∆3∆91^ mice were treated with heparinase followed by SDS-PAGE and western-blot analysis. As a positive control, we utilized a BM matrix extracted from Engelbreth-Holm-Swarm mouse sarcomas (Matrigel^®^) [33, 34], which contains perlecan together with other BM proteins. Heparinase-untreated controls from both the mice brain and Matrigel^®^ did not show any band corresponding to native perlecan (Fig. 1D), possibly due to masking of the epitope by the GAGs and/or the presence of charged GAGs that may prevent perlecan transfer onto a membrane [35, 36]. Compared to untreated controls, the heparinase-treated samples presented a prominent band of the expected molecular weight (Fig. 1D, perlecan core protein being 470 kDa [37]). In the *Hspg2*^∆3∆91^ samples, the perlecan core protein was detectable in western-blot analysis without heparinase and its mobility in SDS-PAGE did not differ between the heparinase-treated and non-treated samples (Fig. 1D), confirming that the core protein in the *Hspg2*^∆3∆91^ mice lacked GAG chains, as expected.

To investigate whether GAG chain deletion affects the localization of perlecan core protein in the perivascular regions, we performed immunofluorescence analyses of brain sections using Col IV and perlecan antibodies (Fig. 1E). Perlecan immunostaining co-localized with Col IV and its distribution was not affected in the *Hspg2*^∆3∆91^ mice as compared to *Hspg2*^+/+^ (Fig. 1E-F). Quantification of the immunoreactive area of Col IV, which marks the BMs of blood vessels, did not show any significant changes in the *Hspg2*^∆3∆91^ mice (Fig. 1G). Altogether, these results showed that the deletion of ex3 and ex91 resulted in GAG chain deficiency in *Hspg2*^∆3∆91^ mice but did not alter *Hspg2* gene expression, core protein localization in the PVS, or the viability of the *Hspg2* mice.

### Absence of Perlecan glycosaminoglycan chains delays perivascular inflow but does not affect the perivascular penetrance of solutes

**Fig. 2:**
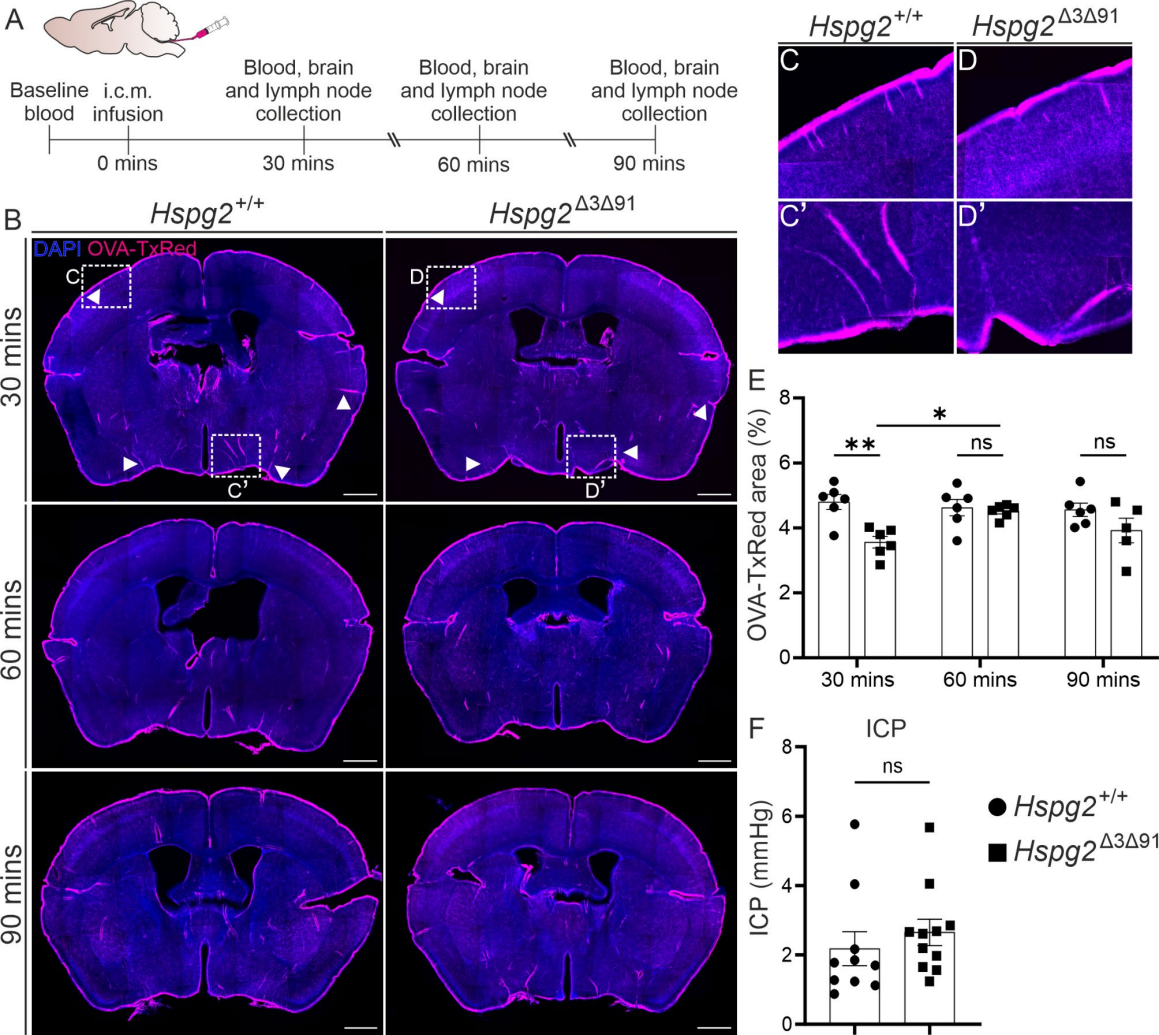
**Deletion of glycosaminoglycan chain attachment sites from the perlecan core protein delays cerebrospinal fluid influx into the brain**. **A**) Schematic timeline for the experiment and sample collection. **B**) Coronal sections of mice brains for the time points indicated, showing the presence of OVA-TxRed tracer (magenta) in the perivascular spaces of the Hspg2^+/+^ and Hspg2^∆3∆91^ mice. White arrowheads indicating the locations with reduced perivascular influx in Hspg2^∆3∆91^ as compared to Hspg2^+/+^. Enlarged images of brain sections highlighting reduced tracer (magenta) in dorsal (D) and ventral (D’) locations in Hspg2^∆3∆91^. **E**) Quantification of the tracer area showing a statistically significant difference between Hspg2^+/+^ and Hspg2^∆3∆91^ at 30 min (*n* = 6) but not at 60 min (*n* = 6) or 90 min (*n* = 5–6 mice per genotype). Intracranial pressure (ICP) measurements did not show any difference between the Hspg2^+/+^ (*n* = 10) and Hspg2^∆3∆91^ (*n* = 11) mice. Scale bar, 1 mm (**B**). The statistical tests used were 2-way ANOVA followed by Sidak’s post-hoc test (**E**) and the Mann-Whitney unpaired two-tailed *t*-test (**F**).  *p < 0.05, **p < 0.01. *ns*, non-significant. Mean ± SEM

To investigate the influence of perlecan GAG chain deletion on the perivascular CSF flow, we injected 2-3-month-old mice with fixable tracer ovalbumin-Texas Red (OVA-TxRed) and measured the area fraction of the tracer in brain sections collected at certain time points (Fig. 2A). Perivascular CSF influx, evaluated based on the area covered by the tracer, was reduced in the *Hspg2*^∆3∆91^ mice relative to the *Hspg2*^+/+^ at 30 min after infusion (Fig. 2B, E). This reduction was more prominent in the ventral aspect (Fig. 2C', D') of the brain than in the dorsal aspect (Fig. 2C, D). Analysis of other time points, however, did not yield any significant alterations in perivascular tracer influx between the *Hspg2*^∆3∆91^ and *Hspg2*^+/+^ mice (Fig. 2B, E). Interestingly, we did observe a significant increase in tracer coverage in the *Hspg2*^∆3∆91^ animals between 30 min and 60 min (Fig. 2E), with values comparable to *Hspg2*^+/+^. No additional increase was observed at 90 mins, suggesting a delay in fluid influx. Importantly, this delay in fluid influx was not a result of pressure changes between *Hspg2*^Δ3Δ91^ and *Hspg2*^*+/+*^ as we observed no significant difference in the intracranial pressure (ICP) (Fig. 2F). Moreover, changes in tracer area in the *Hspg2*^∆3∆91^ mice were not a result of altered AQP4 expression (Supplementary Fig. 1), which has been shown to be critical for perivascular flow [1, 38].

**Fig. 3:**
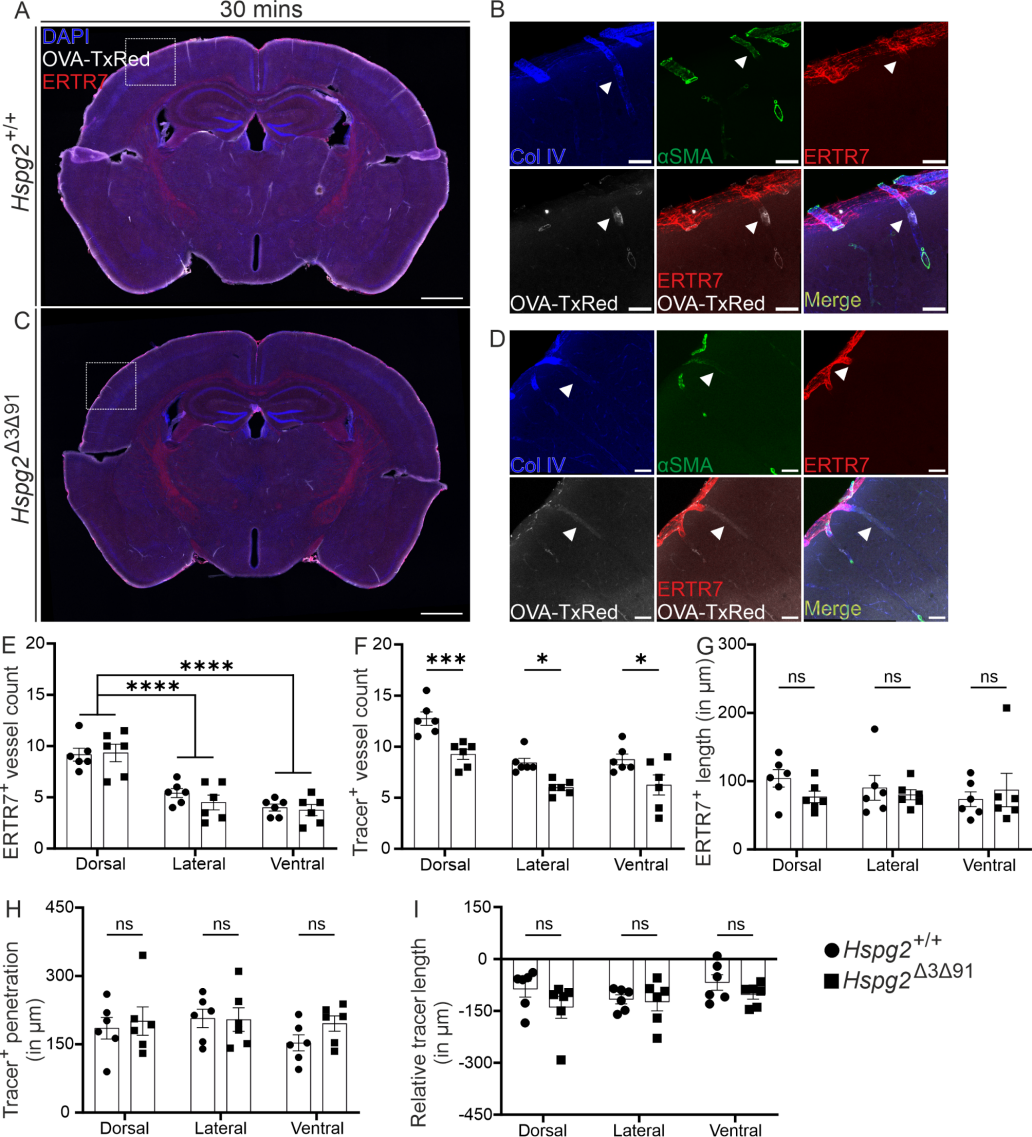
**Penetrating vasculature in the Hspg2**^**∆3∆91**^** mice**. A, C) Brain sections from mice euthanized 30 min after OVA-TxRed tracer (white) infusion into the cisterna magna were co-stained with ERTR7 (red), Col IV (blue) and aSMA (green). B, D) Enlarged insets from A and C respectively, showing the distribution of tracer (white) and its co-localization with ERTR7 and Col IV^+^aSMA^+^ vessels (arteries, white arrowhead). E, F) Numbers of ERTR7 and tracer-positive vessels and their distribution based on location in Hspg2^+/+^ (n=6) and Hspg2^∆3∆91^ (n=6) mice. G-I) Quantification of penetration lengths of ERTR7, tracer, and their relative distance along with their distribution based on their location in the Hspg2^+/+^ (n=6) and Hspg2^∆3∆91^ (n=6) mice. Scale bar; 1 mm (A, C), 50 µm (B, D). The statistical test used was 2-way ANOVA followed by Sidak’s post-hoc test (E-I). *p < 0.05, ***p < 0.001, ****p < 0.0001. ns, non-significant. Mean ± SEM

To analyse further the tracer penetrance along the penetrating arteries, we measured the distribution of tracer fluorescence with respect to the previously described pial cell marker ERTR7 [4, 5] 30 min after tracer infusion. ERTR7-positive cells have been observed lining a fluid-filled space that extends into the brain parenchyma along the penetrating arteries [4, 5]. For this purpose, we used Col IV to label the vascular BM and considered Col IV-positive areas in the brain parenchyma which did not overlap with ERTR7 as BM filled PVS (Fig. 3A-D). As reported before [5], we observed a significant difference in ERTR7-positive vessel coverage between the dorsal and ventral/lateral parts of the brain in both genotypes (Fig. 3E), the former having higher coverage. However, there were no differences in ERTR7-positive vessel coverage between the genotypes (Fig. 3E), nor did we observe any significant differences between the genotypes in the penetration depth of the tracer from the regions where the ERTR7-positive labelling terminated (Fig. 3I). This result suggests that, despite the reduced presence of the tracer (Fig. 3F), the penetration depth of the tracer along the BM was not affected in the *Hspg2*^∆3∆91^ mice (Fig. 3H, I).

To investigate whether the CSF fluid dynamics along the pial arteries could explain the observed difference in tracer inflow kinetics, we infused 500 nm fluorescent beads into the CSF and measured their speed in the PVS of the middle cerebral artery. No differences were noted between the *Hspg2*^Δ3Δ91^ and *Hspg2*^*+/+*^ mice (Supplementary Fig. 2), suggesting that the low resistance periarterial conduct pathways of the CSF are not affected by the removal of GAG chains from the perlecan. Consistent with previous findings [3, 39], no fluorescent microspheres were found surrounding the penetrating arteries.

**Fig. 4:**
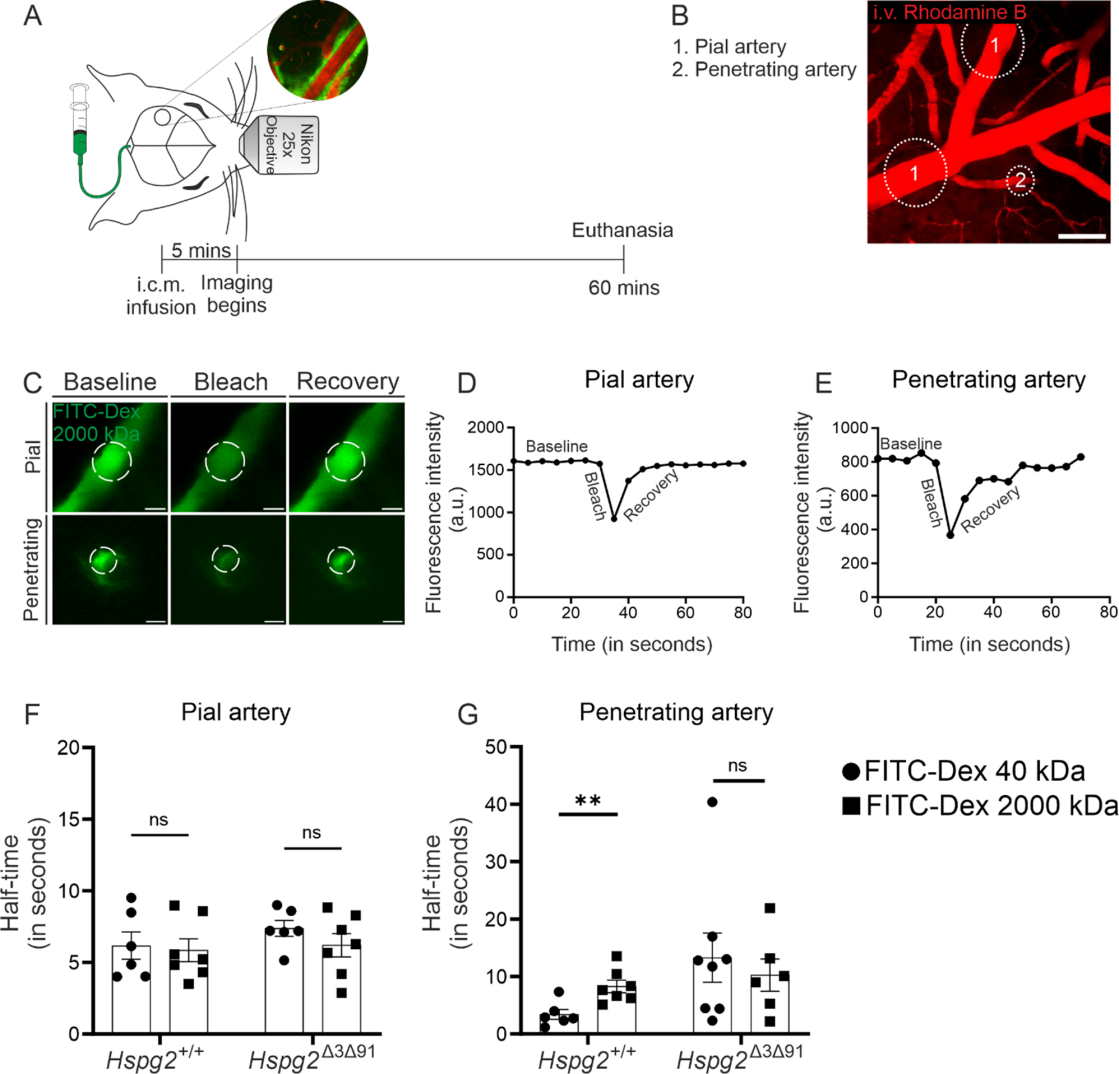
**Kinetics of fluorescent tracers of differing molecular weights in the perivascular spaces of penetrating and pial arteries in the Hspg2**^**+/+**^** and Hspg2**^**∆3∆91**^** mice. A**) Schematic setup with the location of the cranial window (circle) and the timeline of the imaging experiment. **B**) Image of cortical blood vessels (red, i.v. rhodamine). **B**) with the locations (1 and 2) of the bleaching experiment. **C**) Series of images showing baseline, bleach and recovery of the tracer (green), when injected via the cisterna magna, around the pial and in the penetrating vessels. **D-E**) Plots indicating a faster recovery of fluorescence post bleaching next to the pial artery and less steep recovery around the penetrating artery. **F**) Analysis of the half-time of recovery, showing no significant difference between tracers of different molecular weights (FITC-40 kDa and FITC-2000 kDa) around the pial vessels in the Hspg2^+/+^ (*n* = 6–7) and Hspg2^∆3∆91^(*n* = 6–7) mice. **G**) Analysis demonstrating a significant increase in the half-time of the recovery of FITC-2000 kDa tracer as compared to FITC-40 kDa around the penetrating arteries in the Hspg2^+/+^ (*n* = 6–7) mice but not in Hspg2^∆3∆91^(*n* = 6–8). Scale bar, 50 μm (**B**,** C**). The statistical test used was the multiple unpaired two-tailed t-test with Welch’s correction followed by Benjamini and Hochberg correction for multiple t-tests (**F**, **G**). **p < 0.01. ns, non-significant. Mean ± SEM

We then analysed the periarterial solute dynamics along the penetrating arteries (Fig. 4A, B) using FITC-Dextran of different molecular weights, since these had been shown previously to be distributed along the penetrating arteries [1, 39]. Fluorescence recovery after photobleaching (FRAP) was performed on the tracer-filled perivascular compartments of penetrating and pial arteries to compare their tracer kinetics (Fig. 4C-E). No differences in fluorescence recovery time emerged between the tracers of different molecular weights in the pial arteries (Fig. 4F), which is consistent with the convective flow hypothesis [39]. Similarly, the genotypes did not show any differences in fluorescence recovery times, thus supporting the result on perivascular flow of fluorescent beads. In the penetrating arteries of *Hspg2*^*+/+*^ (at a depth of 100 μm), in contrast, half-time of fluorescence recovery was significantly different between various molecular weight tracers (Fig. 4G), suggesting the presence of diffusive rather than convective flow. In the *Hspg2*^∆3∆91^ mice, this diffusive characteristic was disrupted (Fig. 4G), which could plausibly explain the delayed fluid influx observed in the ex vivo analysis (Fig. 2E).

Altogether these results thus suggest that a perlecan GAG chain deficiency delay the solute inflow and alter the diffusive characteristics of solute movement in the BM of the penetrating arteries.

### Solute clearance from the brain parenchyma in *Hspg2*^∆3∆91^ mice

**Fig. 5:**
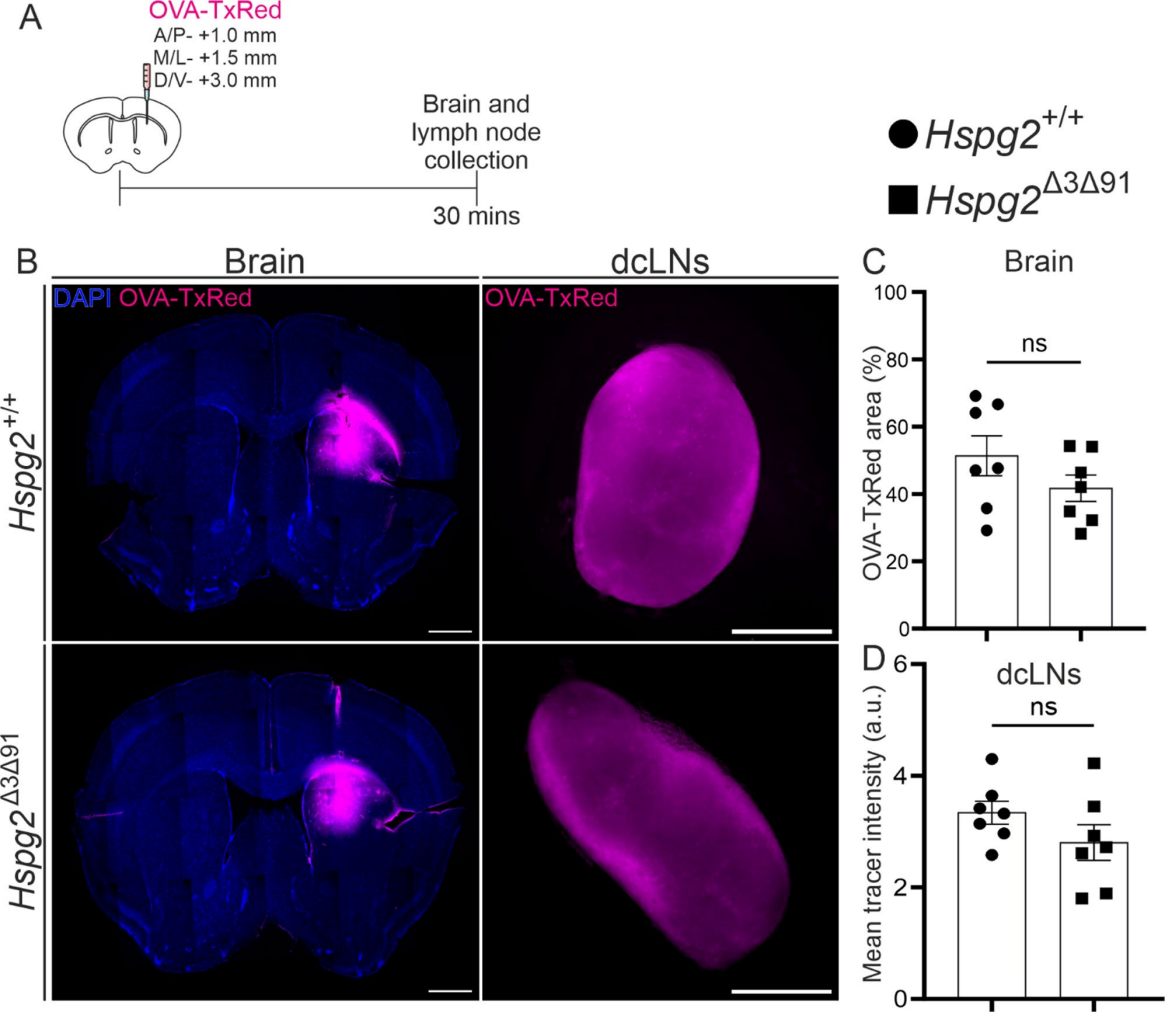
Clearance of ovalbumin-Texas Red tracer (45 kDa) from the brain parenchyma into the deep cervical lymph nodes. **A**) Schematic timeline of tracer infusion into the brain parenchyma along the anterior/posterior (A/P), medial/lateral (M/L) and dorsal/ventral (D/V) axes and collection of the tissue samples. **B**) Illustrative images of the brain and deep cervical lymph nodes (dcLNs) from Hspg2^+/+^ and Hspg2^∆3∆91^ mice, with quantification (**C-D**, *n* = 7 mice per genotype). Scale bar, 1 mm (brain), 500 μm (dcLN). The statistical test used was the unpaired two-tailed t-test with Welch’s correction (**C**,** D**). ns, non-significant. Mean ± SEM

**Fig. 6:**
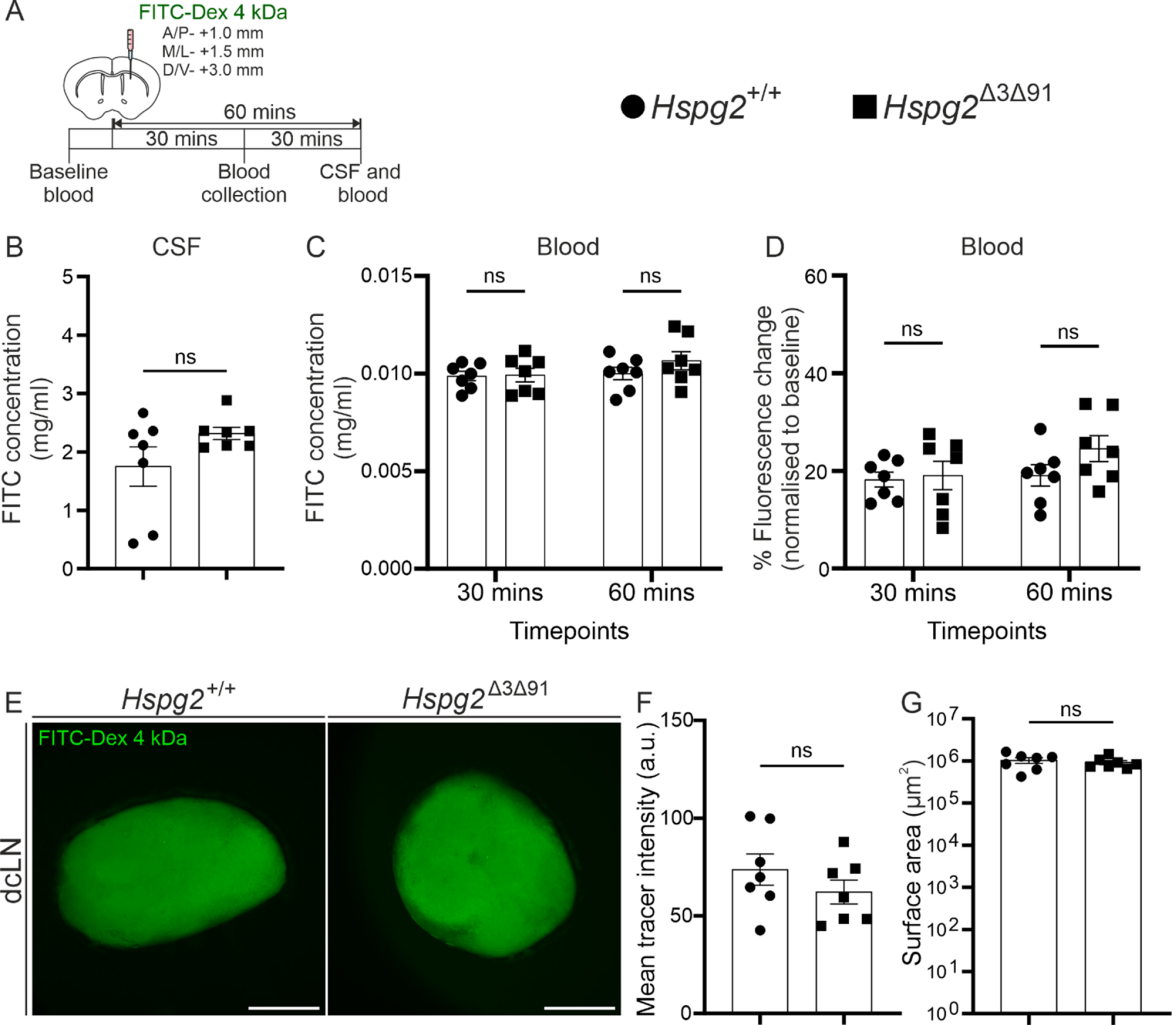
**FITC-Dextran (4 kDa) tracer dye clearance from the brain parenchyma into the cerebrospinal fluid, blood and deep cervical lymph nodes. A**) Schematic timeline of tracer infusion into the brain parenchyma along the anterior/posterior (A/P), medial/lateral (M/L) and dorsal/ventral (D/V) axes and the collection of blood, cerebrospinal fluid (CSF) and tissue samples. **B**) Quantification of tracer concentrations in CSF from the Hspg2^+/+^ (*n* = 7) and Hspg2^∆3∆91^ (*n* = 7) mice. **C, D**) Tracer concentration or its change relative to baseline in blood samples in the Hspg2^+/+^ (*n* = 7) and Hspg2^∆3∆91^ (*n* = 7) mice. **E**) Deep cervical lymph nodes (dcLNs) collected from Hspg2^+/+^ and Hspg2^∆3∆91^ mice 60 min after FITC-dextran 4 kDa injection. **F, G**) Quantification of tracer intensity and size of dcLNs in Hspg2^+/+^ (*n* = 7) and Hspg2^∆3∆91^(*n* = 7) mice. Scale bar, 500 μm (**E**). The statistical tests used were the Mann-Whitney unpaired two-tailed t-test (**B**,** G**), the unpaired two-tailed t-test with Welch’s correction (F) and 2-way ANOVA followed by Sidak’s post-hoc test (**C**,** D**). ns, non-significant. Mean ± SEM

The glymphatic theory suggests that the perivascular compartments act as fluid conduits for the influx of CSF and the clearance of brain interstitial fluid (ISF) that contains metabolites [1]. To investigate the influence of GAG chain removal on brain ISF clearance, we injected mouse brains with OVA-TxRed at the age of 2–3 months (Fig. 5A). The *Hspg2*^∆3∆91^ mice did not display any differences in tracer spreading in the brain parenchyma over 30 min (Fig. 5B, C). Deep cervical lymph nodes (dcLNs) have been described as the primary drainage site of the CSF [40–42] as well as brain derived metabolites [41, 43]. Quantification of the OVA-TxRed tracer amount in dcLNs did not yield any significant difference between the *Hspg2*^∆3∆91^ and *Hspg2*^+/+^ mice (Fig. 5B, D). Previous studies have shown that brain-derived solutes enter the CSF before it drains into the dcLNs via the lymphatic vessels in the meninges [43–46]. Additionally, clearance of brain solutes such as Aβ has been shown to occur across the blood-brain barrier (BBB) via transporters such as Lrp1 or p-gp [47–49]. To analyse further whether the solute clearance routes from the brain are affected in *Hspg2*^∆3∆91^ mice, we injected a small molecular weight tracer (FITC-Dex 4 kDa) into the striatum followed by CSF and blood sampling for fluorescence quantification (Fig. 6A). In 30 min the amount of tracer in the blood increased by ~ 20% (normalized to the baseline) without any further increase at 60 min (Fig. 6D). There were no differences in tracer in the blood between genotypes (Fig. 6C-D). CSF sampling at 60 min confirmed that similar amounts of tracer enter the CSF from the brain in both *Hspg2*^+/+^ and *Hspg2*^∆3∆91^ mice (Fig. 6B). In addition to the CSF and blood, the tracer was found in dcLNs in similar amounts in both genotypes (Fig. 6E), thus suggesting that altering the GAG content of the BM does not affect the clearance of solutes from the brain.

We also analysed dural LVs using the LYVE1 marker and found no significant changes around the confluence of the sinus (COS) area in the *Hspg2*^∆3∆91^ mice (Supplementary Fig. 3). The lack of changes in the LVs was further reflected by the absence of alterations in the drainage of the CSF-injected tracer (Supplementary Fig. 3).

Altogether, this dataset showed that the clearance of solutes from the brain primarily occurs via the CSF, and that this pathway, along with the drainage of solutes from the cranium, remains unaltered in *Hspg2*^∆3∆91^ mice.

### Loss of Perlecan glycosaminoglycan chains results in reduced amyloid beta deposition around the pial arteries

**Fig. 7:**
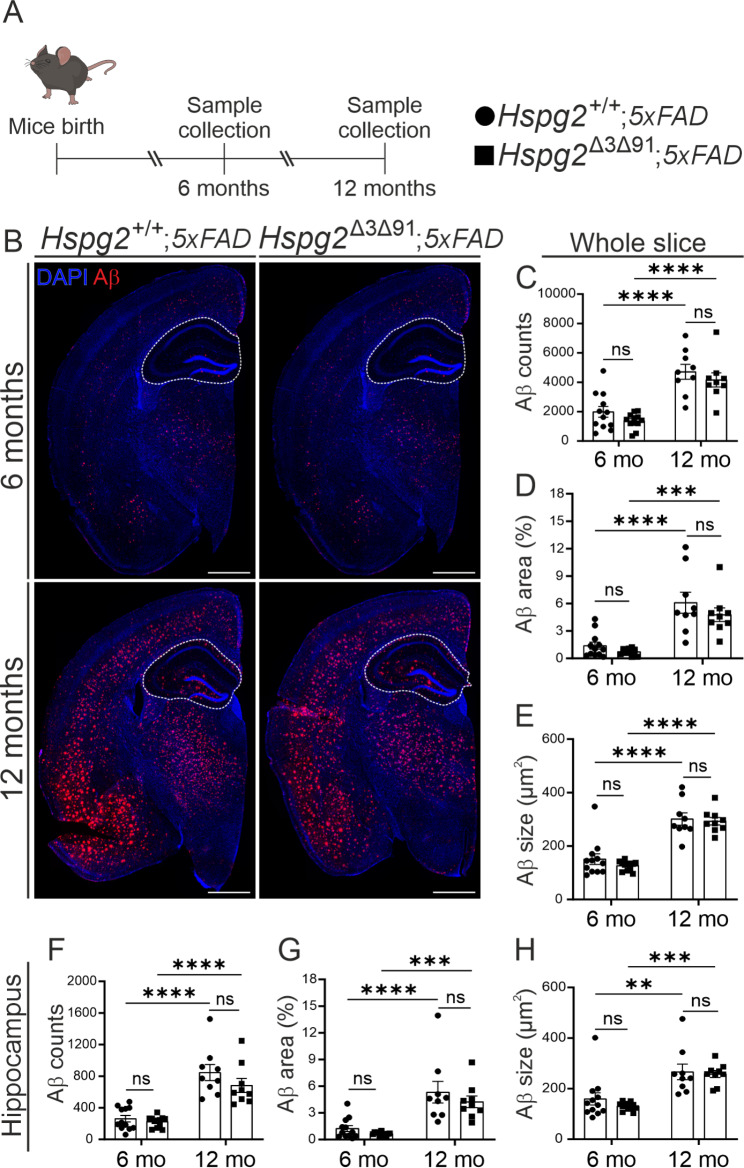
**Hspg2**^**∆3∆91**^**;5xFAD mice show no alterations in amyloid-β (Aβ) load in the brain parenchyma. A**) Timeline of the experiment showing the timing of sample collection. **B**) Images of brain sections (Aβ, red) from the Hspg2^+/+^;5xFAD and Hspg2^∆3∆91^;5xFAD mice at 6 months (*n* = 12) and 12 months (*n* = 9) of age. **C-E**) Quantification of Aβ (red) in the whole brain section. **F-H**) Quantification of Aβ (red) in the hippocampus (white dashed line). Scale bar, 1 mm (**B**). The statistical test used was 2-way ANOVA followed by Sidak’s post-hoc test (**B-G**). *p < 0.05, **p < 0.01, ***p < 0.001, ****p < 0.0001. ns, non-significant. Mean ± SEM

Previous studies have linked impaired solute clearance along proposed perivascular routes to the accumulation of Tau and Aβ [44, 50–53]. Interestingly, HSPGs have been shown to co-localize with congophilic plaques in human brain tissues [16, 54]. To test the importance of perlecan GAGs in Aβ clearance, we crossed the *Hspg2*^Δ3Δ91^ mice with the *5xFAD* AD model (*Hspg2*^Δ3Δ91^;*5xFAD*). Consistent with previous findings [55], the *5xFAD* mice showed an age-dependent increase in Aβ deposition over the whole brain area and separately in the hippocampus (Fig. 7B-H), but no difference in plaque deposition was observed between the *Hspg2*^Δ3Δ91^;*5xFAD* and *Hspg2*^+/+^;*5xFAD* mice at either 6 or 12 months of age (Fig. 7B-H). This finding suggests that the deletion of GAGs from perlecan does not affect amyloid deposition and thus clearance of the brain parenchyma. This finding is further supported by lack of significant changes in expression levels of Aβ in the proteomic analysis of the brain samples from *Hspg2*^Δ3Δ91^ which would suggest an unaltered brain clearance (Supplementary Fig. 1).

**Fig. 8:**
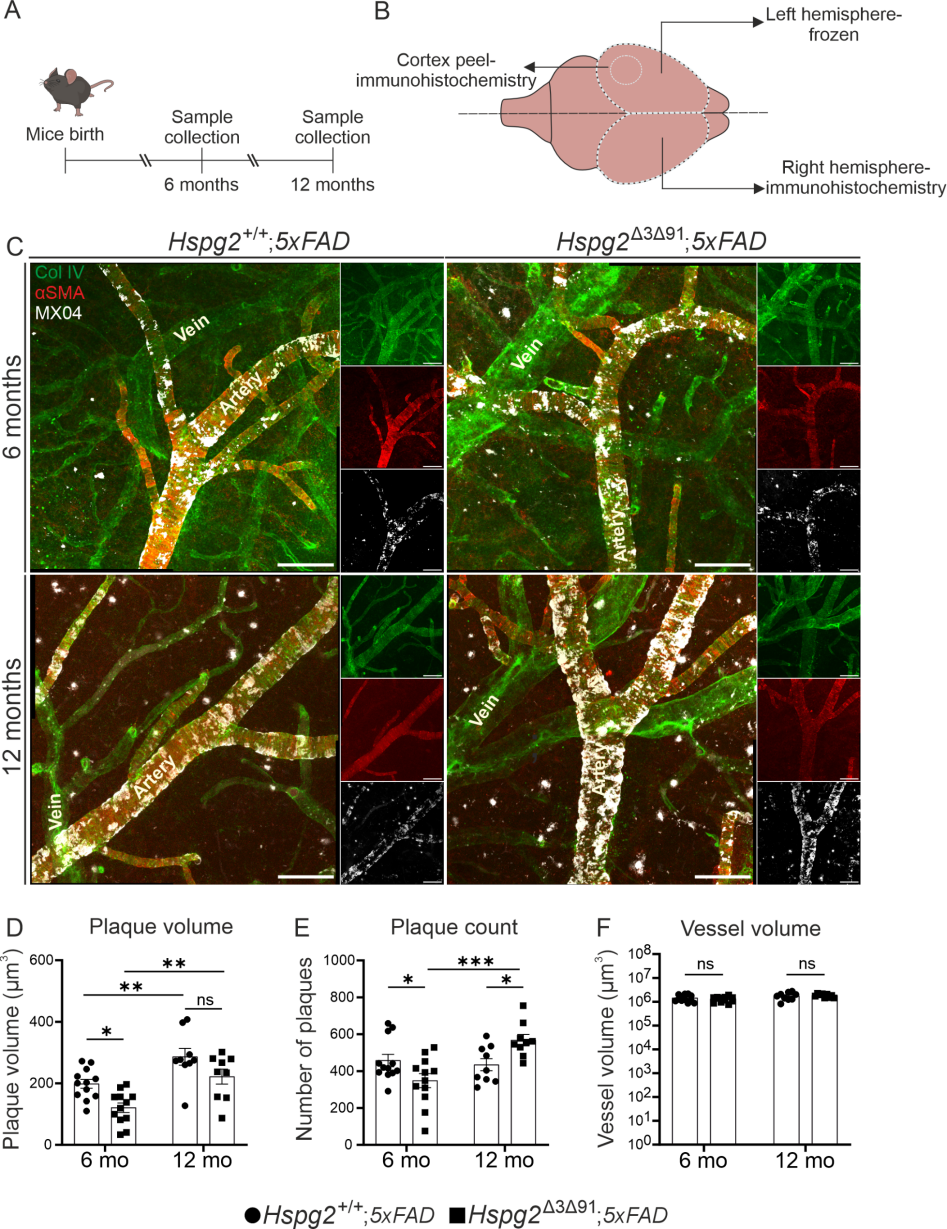
**Reduced amyloid burden around the pial arteries in 6-month-old Hspg2**^**∆3∆91**^**;5xFAD mice. A**) Timeline of the experiment showing the timing of sample collection. **B**) Schematic of sample collection strategy. **C**) Images of the pial artery stained with Col IV (green) and αSMA (red) in the mice at the ages indicated. The amyloid beta (Aβ) plaques were stained using methoxy-X04 (MX04, white). **D-E**) Quantification of the volume and number of MX04^+^ Aβ plaques surrounding the pial vessels shows significantly fewer Aβ plaques in the Hspg2^∆3∆91^;5xFAD mice at 6 months (*n* = 12) than at 12 months (*n* = 9). **F**) The volume of the pial arteries shows no significant differences between the genotypes at timepoints indicated. Scale bar, 100 μm (**C**). The statistical test used was 2-way ANOVA followed by Sidak’s post-hoc test (**D-F**). *p < 0.05, **p < 0.01, ***p < 0.001. ns, non-significant. Mean ± SEM

Previous findings have demonstrated dysregulated BM composition and altered Aβ deposition in the vascular BM in aged and in heparanase-overexpressing transgenic mice [56, 57]. Given the absence of changes in the brain parenchyma, we next examined the potential impact on the perivascular deposition of Aβ in the left parietal cortex, and particularly in the pial vessels (Fig. 8A, B), which had been primarily affected by amyloid load in a previous study [58]. Based on strong alpha smooth muscle actin (αSMA) staining, arteries were distinguished from the veins (αSMA^−^). Consistent with previous findings in the *5xFAD* mouse model [58], Aβ deposition was prominent around the arteries, while the veins were devoid of detectable deposition (Fig. 8C). The *Hspg2*^Δ3Δ91^;*5xFAD* mice showed reduced plaque volume (Fig. 8D) and plaque number (Fig. 8E) around the pial arteries at 6 months of age compared to *Hspg2*^+/+^;*5xFAD* mice. At 12 months of age, the difference in plaque volume was no longer significant, although there was a trend (*p* = 0.07) for reduced plaque volume in the *Hspg2*^Δ3Δ91^;*5xFAD* mice (Fig. 8D). Interestingly, in contrast to plaque volume, the number of plaques at the age of 12 months was significantly higher in the *Hspg2*^Δ3Δ91^;*5xFAD* mice than in their *Hspg2*^+/+^;*5xFAD* counterparts (Fig. 8E), suggesting unstable plaques resulting in a greater number but smaller sized aggregates. The vessel volume did not differ between the genotypes (Fig. 8F), indicating a lack of influence on the plaque volume.

To determine whether the reduced Aβ deposition in the *Hspg2*^Δ3Δ91^;*5xFAD* mice at 6 months of age could be due to altered CSF clearance, we analysed Aβ deposition in the dura mater with co-immunostaining of podocalyxin (highlighting the venous sinus) and an Aβ antibody. The staining showed vessel-associated Aβ deposition with no difference in immunoreactive area between the genotypes (Supplementary Fig. 4). Interestingly, we did observe an age-dependent increase in the plaque area in the *Hspg2*^+/+^;*5xFAD* group (Supplementary Fig. 4). This result suggests that Aβ similarly accumulates in the dura mater, which contains lymphatic vessels and large collecting venous sinuses and serves as a CSF drainage route of solutes [59]. Altogether, the data in *Hspg2*^Δ3Δ91^;*5xFAD* mice indicated that perlecan GAG chains have a role in Aβ aggregation around arteries but not in Aβ clearance from the brain parenchyma.

## Discussion

In this study, we provide evidence of the contribution of perlecan GAG chains on solute movement and brain clearance. In contrast to the detrimental effect of perlecan deletion on development and viability [28, 29], deletion of the GAG attachment sites appears to be well adapted, causing relatively minor defects [60, 61]. Upon complete removal of GAG chains, our proteomic and transcriptomic analysis, along with the immunofluorescence data, revealed no significant changes associated with the brain ECM, further supporting previously published findings in which only N-terminal GAG attachment sites were deleted [60]. Also, we did not observe any changes in the parenchymal border macrophages, which have been shown in a previous study to control ECM remodelling [62]. Instead, we found that the complete removal of GAG chains from perlecan had a transient effect on CSF influx in mice as defined by the smaller number of tracer-positive PVSs in the brain cortex and changes in the diffusivity characteristics of the perivascular BM.

The glymphatic hypothesis states that fluid flows into the brain parenchyma along the PVS [1]. PVS around the pial arteries may provide a relatively low-resistan*ce* pathway [3] between the brain cortex and the subarachnoid space, while there may be higher resistance around the penetrating arteries and arterioles (following the merging of pial layers with the vessel wall [5]) and capillaries, where the PVS is filled with BM [4]. The perivascular movement is probably regulated more in this compartment than in the PVS of the pial arteries, and may occur based on the size, shape, binding and the charge of solutes and composition of the BM. Accordingly, 1 μm fluorescent beads injected into the CSF are reported to be confined to the superficial aspects of the brain [3]. In another study, the penetration of 150 kDa immunoglobulin molecules was found to be more restricted than that of ~ 17 kDa single domain antibodies [6]. The importance of the perivascular BM for the regulation of solute movement was assessed in a more recent study in which CSF access to PVS was hindered in mice with abnormal accumulation of BM proteins (Col IV and laminin) in the penetrating arteries as a result of depletion of the ECM remodelling parenchymal bordering macrophages [62]. The GAG chains of HSPGs in BM provide the hydrogel with a net negative charge and may thus contribute to the permeability and hydration status of the BM. Our FRAP analysis suggested that diffusion contributes to the dynamics of fluorescent tracers around the penetrating arteries/arterioles and that deletion of the perlecan GAG chains affected that characteristic. This does not, however, explain the delayed inflow in *Hspg2*^Δ3Δ91^ mice, as the penetrance of the tracer from the ERTR7-defined perivascular compartment onwards was similar between the genotypes. It is thus possible that the altered periarterial diffusion found in *Hspg2*^Δ3Δ91^ mice is compensated by improved bulk flow, which has been suggested as the main driver of solute movement along the penetrating arteries [39]. The differences in fluid influx were not accompanied with changes in the expression of the ERTR7-positive pial layer in relation to the penetrating vessels. Moreover, no differences were observed in bulk flow along the branches of the middle cerebral artery. It should be noted, however, that multiphoton analysis covers only a very limited region below the parietal bone, and it is still possible that some alteration in speed exists in the more distal branches of the middle cerebral artery. Vasomotor waves and vascular pulsatility have both been shown to contribute to the periarterial flow of solutes [63, 64], and impaired ICP has also been shown to influence periarterial transport [65]. However, in *Hspg2*^Δ3Δ91^ mice the ICP was not altered. It is thus plausible that there may exist local variations in arterial function/cerebral autoregulation in *Hspg2*^Δ3Δ91^ mice which could account for the observed transient change in tracer influx.

In addition to serving as an inflow route, it has been suggested that the vascular BM may provide a clearance pathway of brain-derived solutes [2, 7]. Fluorescent tracers injected in the brain parenchyma rapidly end up in the BM of the arterial walls, from which it is postulated that they are transferred to the CSF [2, 7]. Alternatively, according to the glymphatic hypothesis, solutes in the interstitial space are transported to the perivenous space, where they move to the CSF [1]. These solutes have been shown to enter the CSF [44, 45, 66] and be cleared by the dural lymphatic vessels [41]. We could not observe any alteration in the spreading of the fluorescent tracer in the brain parenchyma of our *Hspg2*^Δ3Δ91^ mice in the same time window as we observed the delay in the inflow of CSF tracers, nor could we see any impaired drainage into the dcLNs or blood. Our results thus suggest that the GAG content of the vascular BMs (where perlecan is exclusively located in the brain) does not contribute to brain clearance, as similar amounts of solutes ended up in the CSF in both genotypes. It is possible that other efflux pathways such as nerve fibre tracts, may play a more significant role in the transfer of solutes from the brain to the CSF, as shown in a previous study [67].

To establish the relevance of altered BM GAG content and solute clearance to pathological contexts, we analysed amyloid accumulation in the brains of *Hspg2*^∆3∆91^;*5xFAD* mice. In AD patients, perlecan has been shown to be localised together with the amyloid fibrils of neuritic plaques and cerebrovascular amyloid deposits, with a possible role in plaque formation [16]. Moreover, perlecan has been found to be essential for the formation and stability of Aβ fibrils in vitro [27] and additional studies have confirmed that the sulphate moieties of GAG chains, or more specifically heparan sulphate (HS) GAG chains, play a crucial role in accelerating the formation of the fibrillar form of Aβ [27, 68]. Furthermore, neuron specific deletion of *Ext1*, which led to a lack of the enzyme required for GAG chain elongation, also led to a reduced Aβ load in mice [51]. We found that perlecan GAG deficiency does not influence amyloid deposition in the brain, thus further supporting the finding of unaltered clearance of fluorescent tracers. Interestingly, the *5xFAD* mouse model displayed little to no amyloid deposition in the vascular walls of the brain parenchyma, while the pial arteries were extensively decorated with amyloid deposits [58]. We observed smaller-sized and less numerous Aβ plaques around the pial arteries in the *Hspg2*^∆3∆91^;*5xFAD* mice at 6 months of age, while no change was observed in the clearance kinetics of differently sized fluorescent tracers from CSF of young mice. This suggests that a reduction of HS GAG chains rather than impaired clearance may contribute to amyloid deposition in the arterial walls, a finding that is supported by in vitro studies [27, 68]. An in vitro study has also demonstrated that the presence of perlecan HS GAG chains enhances Aβ_1−40_ fibril formation at early time points [27], and interestingly, it also found that the stability of the Aβ_1−42_ isoform [27], which is considered to be the main isoform driving aggregation [69, 70], was reduced in the absence of perlecan HS GAG chains. In *5xFAD* mice, the CSF concentration of the aggregation-prone Aβ_1−42_ isoform declines markedly with age while the concentration of the Aβ_1−40_ isoform remains unaltered [71]. The favoured isoform profile in aged *5xFAD* mice is Aβ_1−40_ [71], fibrillization of which is only transiently influenced by HS GAG chains in vitro [27]. This may explain why the reduced HS GAG content in the *Hspg2*^∆3∆91^ mice had less influence on amyloid deposition in the case of 12-month-old mice than in those aged 6 months. Interestingly, recombinant perlecan domain V, which contains C-terminal GAG binding sites in exon 91, has been shown to inhibit Aβ-induced cytotoxic effects in cultured hippocampal and cortical neurons [72, 73] and endothelial cells [74]. Thus, while the GAG chains of perlecan promote the stability of Aβ fibrils in vascular walls, they may simultaneously reduce the cytotoxic effects of Aβ. The role of HS GAG chains as a potential therapeutic target for regulating amyloid deposition in the walls of cerebral arteries thus clearly warrants further investigation.

## Conclusions

The PVS surrounding cerebral blood vessels serves as a pathway for fluid and solute inflow and outflow, thereby contributing to brain clearance. At the level of the perforating arteries/arterioles, the PVS is formed of BM, although it has not been known how transport along and through this BM is facilitated. To address the importance of perlecan as a major perivascular BM proteoglycan, we generated a novel mouse line *Hspg2*^∆3∆91^, lacking N- and C-terminal GAG attachment sites from the perlecan core protein. For the first time, we demonstrate that perlecan GAG deficiency affects the solute movement and diffusion in the PVS of the penetrating arteries but not their clearance from the brain parenchyma. The results additionally showed that perivascular BM GAG chains have a role in Aβ plaque formation on the walls of pial arteries. Thus, our findings also open up avenues for future research in targeting GAG chains as a therapeutic measure for reducing Aβ aggregation and alleviating AD pathology.

## Materials and methods

### Generation of the *Hspg2*^∆3∆91^ mouse line

The targeting vector for *Hspg2* exon 91 deletion (*Hspg2*^Δ91^) was first generated by the recombineering approach described previously [75]. Briefly, genomic BAC-clones RP23-345J3 overlapping the *Hspg2* gene locus were obtained from BACPAC resources (Children’s Hospital Oakland). Bacterial cells carrying these BAC-clones were electroporated with pSC101-Bad-gbaA vector and a floxed *neo*-cassette with homologous arms (5′-cggctcctcccatgtccatcaggaagctcatagctgcctatgact-loxp-PGK-EM7-Neo-loxP-gcttccattggatcccctgggacaggagttatagggagctgtaca-3′) was electroporated into the same bacterial cells to replace the exon 91. Next, a gene targeting vector was cloned into the p15A-amp-HSV-DTA-rpsL-BSD plasmid. Two homologous arms were generated by PCR using a P15A-amp-HSV-DTA-rpsL-BSD plasmid as the template and the primers: 5′-gaaccaggttccctggctgcgccgaggtatgttctggaattatggtggcatatggaggacgtcgacaaatcaccggtgacccgggtc-3′ and 5′-ggacctgactaaggggtaagcaggcccagactgtggctcttgggtcctagagggaaccccaaatcgccggcgacttaagtc-3'. The PCR-fragment with its homology arms was electroporated into bacterial cells having BAC-clones with a *Neo*-cassette in the *Hspg2* exon 91 and colonies were confirmed by sequencing. The SalI-linearized targeting construct was electroporated into B6 mouse embryonic stem (ES) cells and selected with G418. Genomic DNA isolated from resistant colonies was screened by PCR. Mutation in the resulting *Hspg2*^Δ91^ mouse line was confirmed by sequencing cDNA from several tissues. The *Neo*-cassette was removed from the *Hspg2*^Δ91^ allele by crossing with the Cag-Cre mouse line. Isolation and expansion of ES cells (homozygous for Δ91) was performed using the 2i method [76].

The targeting vector for *Hspg2* exon 3 deletion (Δ3) was generated by the recombineering approach [75]. Briefly, genomic BAC-clones RP23-345J3 overlapping the *Hspg2* gene locus were obtained from BACPAC resources (Children’s Hospital Oakland). Bacterial cells carrying BAC-clones were electroporated with the pSC101-Bad-gbaA vector and a *Cre-Neo*-cassette with homologous arms flanking the third exon (5′-tcaggccatacgctatgatggaggcctccaagctaactaggcatggggtgtgggtgggg-loxp-PGK-EM7-Neo-loxP- tcagttcaactgatgggccctaagcctccagcctcagctctgcgcggctggctgcccttggggtgtggc−3′) was electroporated into the same bacterial cells to replace the exon 3. Next, a targeting vector was cloned into the p15A-amp-HSV-DTA-rpsL-BSD plasmid. Two homologous arms were generated by PCR using P15A-amp-HSV-DTA-rpsL-BSD plasmid templates and the primers: 5′-tgccagcaggtcctcatcatcagagaggtatgagtaggtccagccataccggctcgctgtttaccaatgcttaatcagtgaggc−3′ and 5′-tgccccagttcccaagagtctgcacggagacggagtttgcttgccacagctataatgagtgtcgacttaataagatgatcttcttgagatcg−3′. Then the PCR-fragment with its homology arms was electroporated into bacterial cells having BAC-clones with the *Neo*-cassette in the *Hspg2* exon 3 locus and colonies were confirmed by sequencing. The SalI-linearized targeting construct was electroporated into the resulting exon 91-deleted ES cells and selected with G418. Genomic DNAs isolated from resistant colonies were screened by PCR to identify ES cells having exon 3 and 91 deletions in the same allele. Correctly targeted ES clones were used for blastocyst injection to generate the *Hspg2*^Δ3Δ91^ mouse line. The presence of *Hspg2*^Δ3Δ91^ deletion was confirmed at the cDNA level from several tissues by sequencing the exon 3 and exon 91 regions.

### Animals and housing

All the experiments were performed on adult (2–3 months of age) *Hspg2*^Δ3Δ91^ mice and *Hspg2*^+/+^ littermates. Heterozygote *5xFAD* mice (Jackson Laboratories) were crossed with *Hspg2*^Δ3Δ91^ mice to obtain the *Hspg2*^Δ3Δ91^;*5xFAD* mouse line. Age-matched littermates (*Hspg2*^+/+^;*5xFAD*) were used as controls. Both male and female mice were used in all the experiments. All the genetically modified mouse lines used in the experiments were bred and maintained at Oulu Laboratory Animal Centre and kept in a C57BL/6NCrl background (Charles River Laboratories). The animals were housed in a temperature and moisture-controlled room under a 12-light/dark cycle with *ad libitum* access to food and water. The experiments were performed on animals anaesthetized using a cocktail of ketamine (100 mg/kg) and xylazine (20 mg/kg), and lidocaine (10 mg/kg) was injected subcutaneously into the surgical sites 5 min before the procedure.

### Cisterna magna cannulation and infusion

Mice were placed in a stereotaxic frame after checking the depth of their anaesthesia by pedal reflex. The cisterna magna was exposed by gently separating the neck muscles, and BTPE-10 tubing filled with saline (0.9%) and connected to a 30G dental needle was inserted in the cisterna magna and fixed in place with superglue [77]. The tracers were diluted in artificial CSF (Tocris Bio) and infused into the cisterna magna via a connecting line into the cannula using a Hamilton syringe operated with a microinjector (KD Scientific). 4 µl (two-photon imaging) or 10 µl (ex vivo analysis) of tracer was infused at a rate of 1 µl/min or 2 µl/min, respectively. The connecting line was removed after the infusion and the cannula sealed using an electrocauter (Bovie, change a tip DEL1). For ex vivo analysis, animals were kept in a warm chamber until the end of the experiments.

The tracers used for the cisterna magna infusions were: FITC Dextran 40 kDa (FD40S-250MG, Sigma; 50 mg/ml), FITC Dextran 2000 kDa (52471-1MG, Sigma; 50 mg/ml), ovalbumin Texas Red 45 kDa (O23021, ThermoFisher Scientific; 1 mg/ml) and fluorescent microspheres (505/515) 500 nm (F8813, ThermoFisher; 1:4 dilution).

### Femoral vein cannulation

Anaesthetized animals were placed in a supine position on a heat pad to maintain a body temperature of 37 °C. A small incision was made using scissors to expose the femoral vessels, followed by cannulation of the femoral vein with BTPE-50 tubing. Either Rhodamine-B dextran 70 kDa (R9379-250MG, Sigma; 5 mg/ml) or Evans blue (E2129-50G, Sigma; 0.5 mg/ml) was injected intravenously during the in vivo imaging to visualize the blood vasculature.

### Skull thinning and two-photon imaging

Anaesthetized mice were placed on a stereotaxic frame and the head was stabilized using ear bars. The parietal bone was thinned using a dental drill with a 1 mm bit to improve light penetration. A four-wing head plate (Neurotar) was attached to the top of the skull and secured using dental acryl. Electrocardiogram (ECG) and respiration recordings were made by inserting subdermal electrodes and a respiration sensor, respectively, linked to an iWorx data acquisition system (iWorx IX-RA-834). The head was stabilized using a stabilizer (Neurotar) and the animal was placed under the objective. Imaging was performed using Nikon A1R MP + multiphoton and a CFI75 APO LWD 25x/1.10 water immersion objective after identification of the middle cerebral artery, a large-calibre vessel which emerged from the lateral aspect of the skull window and traversed towards the midline. The image data were acquired using a resonant scanner at 30 Hz using 920 nm laser. Time-lapse imaging was performed for 1 min each, as indicated in the experimental timeline. Videos were imported into MATLAB (MathWorks) and bead tracking was performed with the Kalman filter approach [78].

### Fluorescent recovery after photobleaching

Various molecular weight FITC-dextran tracers were injected into the CSF. The focal plane was set to the maximum diameter of the middle cerebral artery and 100 μm from the surface of the vessel for the penetrating arteries. Fluorescence recovery after photobleaching (FRAP) was performed using a 920 nm laser to bleach the circular region of interest (ROI) defined in the PVS containing the tracers. Arteries penetrating the brain parenchyma were identified and a ROI was established in the PVS at a depth of 100 μm from the surface of the vessel. Stimulations that achieved ~ 40–50% bleaching of the baseline fluorescent intensity were used in the analysis, and imaging was performed with a Galvano scanner to follow the recovery of the tracer fluorescence. For analysis, raw files were imported into a Fiji processing package of Image J2 software (Version 1.53, National Institute of Health) and the intensity value of the ROI was exported into Microsoft Excel. The fluorescence change was calculated for each ROI based on the bleached values and using GraphPad Prism 10 (GraphPad Software), non-linear regression with one phase association was performed to calculate the half-time of fluorescence recovery and the average value of half-time of recovery per location was reported.

### Intraparenchymal injection

Anaesthetized mice were placed in a stereotaxic frame and the head was fixed using ear bars. 1 µl of tracer was injected A/P (anterior/posterior) + 1.0, M/L (medial/lateral) + 1.5, D/V (dorsal/ventral) -3.0, relative to the bregma through a 33G needle (7747, Hamilton) at a rate of 0.2 µl/min using microinjector (KD Scientific). The needle was left in place for 4 min after the infusion and slowly retracted to avoid backflow. The tracers used were ovalbumin Texas Red 45 kDa (O23021, ThermoFisher Scientific; 1 mg/ml) and FITC Dextran 4 kDa (46944-500MG, Sigma; 25 mg/ml).

### Intracranial pressure measurement

A 30G dental needle connected to a BTPE-10 tube filled with saline (0.9%) was inserted through the dura overlaying the cisterna magna and secured with superglue. The tubing was connected to a pressure transducer (BP-102) and recordings were made using the iWorx data acquisition system (iWorx IX-RA-834) for 10 min. The data were analysed using Labscribe 4 software and the mean value over 10 min per animal was reported.

### Cerebrospinal fluid collection

CSF was collected at the time points indicated in the experimental timeline. The cisterna magna of anaesthetized mice was exposed and a 30G dental needle attached to BTPE-10 tubing and a Hamilton syringe was used to puncture the dura overlying it. The tubing was held in place with forceps and CSF was withdrawn using a syringe connected to a microinjector (KD Scientific). The CSF was then transferred to a 1.5 ml test tube, frozen in liquid nitrogen and stored at -70 °C until further use. CSF with blood contamination was discarded from further analysis.

### Blood sampling and serum collection

The skin of a hind limb of each anaesthetized mouse was cut, and the underlying femoral vein was exposed. A tiny hole was made in the ascending saphenous vein with a 27G needle and 80–100 µl blood sample was collected as indicated in the experimental timelines. The blood samples were allowed to stand for 30–60 min on ice and then centrifuged at 5000 g for 10 min to separate the serum, which was snap frozen in liquid nitrogen and stored at -70 °C until use.

### Fluorescence analysis

For the quantification of fluorescence tracers in the CSF and blood serum, samples were thawed on ice and diluted (1:20) in saline (0.9%). Samples were pipetted in triplicate on a 96-well plate and signal were measured using a fluorometer (PerkinElmer, VICTOR3V 1420 Multilabel counter) with excitation / emission of 485 nm / 535 nm (25 nm band-pass filter).

### RNA extraction and quantitative PCR

Brain samples were harvested from the mice after transcardial perfusion with 1xPBS and split into two hemispheres. The left hemisphere was snap frozen, sterile metal beads were used to lyse the tissue samples in Qiagen TissueLyser LT and total RNA was isolated using the Fibrous Tissue Mini Kit (Qiagen) following the manufacturer’s protocol. 3 µg of total RNA was used to synthesize cDNA. 5 µl of Brilliant III Ultra-Fast SYBR Green QPCR master mix (600882, Agilent Technologies) with 1 µl of forward and 1 µl of reverse primers were used. 2 µl of cDNA (diluted 1:3 in H_2_O) was used for the reaction. qPCR was performed in duplicates using a CFX96 qPCR instrument (Bio-Rad). *Sdha* was used as a reference gene. The primer sequences are presented in Supplementary Table 1.

### Protein isolation

Brain hemispheres isolated after transcardial perfusion were snap frozen in liquid nitrogen and stored at -70 °C. Samples were lysed in lysis buffer (25 nM Tris-HCl pH 7.6, 1% NP40, 1% sodium deoxycholate, 0.1% SDS in sterile water) containing 1:100 protease inhibitors (P8340, Sigma Aldrich). Protein concentrations were quantified using PierceTM BCA Protein assay kit (23227, Thermo Scientific) and protein samples stored as aliquots at -70 °C.

### Heparinase treatment of brain homogenates

A small aliquot of protein isolated from the brain hemisphere was thawed on ice and the protein amount was re-quantified. An equal amount of protein was taken and 0.02 IU/ml of heparinase I and III (H3917, Sigma) was added to it. 8 µl of Matrigel^®^ (356234, Corning) was mixed with 10 µl of heparinase I and III (H3917, Sigma). For treatment control, 1xPBS was added to all the samples in equal volume. All the samples were incubated at 37 °C for 2 h and further processed for SDS-PAGE and western blotting.

### SDS PAGE and Western blot analysis

A small aliquot of protein was thawed on ice and the proteins were denatured (reduced) by heating at 98 °C for 5 min with 1% β-mercaptoethanol (M3148, Sigma-Aldrich). Proteins were separated with SDS-PAGE using 12% separating polyacrylamide gels and transferred onto a nitrocellulose membrane (Perkin Elmer). Brain and Matrigel^®^ samples treated with heparinase (and their untreated controls) were denatured (reduced) by heating at 37 °C and 98 °C, respectively, for 10 min with 1% β-mercaptoethanol (M3148, Sigma-Aldrich). The samples were separated with SDS-PAGE using self-prepared 4% concentrating and 5% separating polyacrylamide gels and then transferred onto a nitrocellulose membrane (Perkin Elmer). The membranes were blocked in 5% milk powder – 0.05% Tween 20 in 1×PBS for 1 h at room temperature (RT) and incubated with primary antibodies (Supplementary Table 2) diluted in solution A (1% bovine serum albumin and 0.05% Tween 20 in 1xPBS) overnight at + 4 °C. The membranes were then incubated with horseradish peroxidase (HRP)–conjugated secondary antibodies (Supplementary Table 2) for 1 h at RT, and the signals were detected using Lumi-Light Western blotting substrate (Roche) and imaged with a LAS-3000 luminescent image analyser (Fujifilm). The western blot images were analysed with ImageJ software (Version 1.53, National Institute of Health).

### Liquid chromatography - mass spectrometry

A 10 µl aliquot of protein (isolated as described earlier) was mixed with 50 µl acetone, vortexed and incubated overnight at -20 °C. The samples were centrifuged at 15,000 g for 30 min at 4 °C and the pellet was washed with 80% acetone and centrifuged in the same way before further processing by a derivative of the SPEED-method [79]. The protein concentration was estimated by UV absorption using a nanodrop device and about 1 µg of each sample was applied to nano LC-MS.

For data acquisition, Waters nano-Aquity system (Thermo) was operated with a Lumos Fusion Orbitrap mass spectrometer (Thermo) as the detector. Samples were trapped onto a Waters nano ease MZ symmetry 0.18 × 20 mm precolumn and eluted over a nano ease peptide BEH C18 (300Å, 75 μm x 15 cm, 1.7 μm beads (Waters)) analytical column under the following conditions: flow 0.3 µl/min, column temperature 40 °C, gradient from 97% solvent A (0.1 formic acid in water) to 3% solvent B (0.1% formic acid in acetonitrile), followed by 20% solvent B for 65 mins, and increase to 35% solvent B for 31 min before a 14 min increase to 80% solvent B, where it was kept until the next run. MS data were acquired in data-dependent acquisition mode with survey scans at a resolution setting of 120,000, mass range 375–1500, automatic gain control (AGC) 4e5, fill time under 100 msec. MS-MS interrogation was triggered so that multiple charged ions with an intensity above 2e4 were analysed in the orbitrap, while ions between 1e3 and 3e4 were passed into the ion trap. AGC was set to 5e4 with 100 msec maximum and resolution setting of 15,000 for the orbitrap analyses, while the ion trap was filled with AGC 10,000 for a maximum of 300 msec. High energy collision dissociation with 30% collision energy was used for fragmentation in both cases.

Raw data were analysed with Proteome Discoverer 2.2 (Thermo). Sequest was used as a search engine with the following settings: Swiss Prot homo database (v2017-07-05) with trypsin digestion allowing up to 2 missed cleavages, mass accuracy 10 ppm for parent ions, 0.02 Da for fragments analysed by the orbitrap and 0.6 Da for-ion trap data. Carbamidomethylation on cysteine was specified as a fixed modification and acetylation of protein N-termini, deamidation on glutamine and asparagine, and oxidation of methionine were specified as dynamic modifications. Sequest results were passed to the Percolator node with 0.01 and 0.05 set as the limits for the strict and relaxed false discovery rates (FDR), with delta C below 0.05. The same FDRs were used in protein identification for the Peptide Validator and Protein FDR Validator nodes.

### Bulk mRNA sequencing and analysis

RNA isolation was performed for brain tissues from 5 to 6 biological replicates per genotype. RNA samples were sent for sequencing to Novogene (Cambridge UK), and sequencing was performed at a depth of 25–30 million pair reads. FASTQ files with raw sequencing reads were aligned to mm10 genomes using the HISAT2 alignment tool [80]. HTSeq [81] was utilized to sort the reads into feature counts. Counts were then imported into R-Studio (v4.1), and DESeq2 [82] was used to normalize the raw counts and perform differential expression analyses. To correct for the false positives arising from multiple testing, p-values were corrected using the Benjamini-Hochberg method and genes with adjusted p-value < 0.1 were used for downstream enrichment analysis. Enrichment analysis was performed using the gene set collections obtained from MSigDB [83]. Packages such as ggplot2 [84] and pheatmap were used for plotting the graphs.

### Tissue harvesting and processing

The anaesthetized mice were euthanized either by cervical dislocation or transcardial perfusion using 1xPBS. The skin around the head was removed and a fine scissor was inserted in the cisterna magna to cut the dura along the parietal bone until the rostral part was reached from both sides. The top of the skull was carefully lifted, dropped into 4% paraformaldehyde (PFA) and incubated overnight at + 4 °C. The brain was lifted from the bottom and isolated by removal of the optic chiasm. Brain samples for the drainage (mice with i.c.m. injections) or efflux (mice with intraparenchymal injections) experiments were fixed overnight at + 4 °C in 4% PFA before being transferred to 1xPBS with Azide (0.02%) until further processing.

The brains from the AD mice were isolated after perfusion with 1xPBS and dissected into halves using a brain matrix. A small portion of the parietal lobe from the left hemisphere was cut transversely using fine scissors and fixed in 4% PFA overnight at + 4 °C, while the remainder of the left hemisphere was snap frozen using liquid nitrogen and stored at -80 °C for future use. The right hemisphere was fixed in 4% PFA overnight at + 4 °C. The base of the skull attached to the spinal cord was isolated and fixed in 4% PFA overnight at + 4 °C. All the samples were transferred to 1xPBS with Azide (0.02%) after fixation. The fixed brain tissues were cut into either 50–100 μm sections using a vibratome (Leica VT1200). The sections ranging from the bregma A/*P* + 1.8 to -3.0 were collected in 1xPBS and stored at + 4 °C in 1xPBS with 0.02% sodium azide.

Both the superficial cervical lymph nodes (scLNs) and the dcLNs were isolated in all the experiments and fixed overnight in 4% PFA at + 4 °C before being transferred to 1xPBS with Azide (0.02%) for long-term storage.

### Immunohistochemistry

After the i.c.m injections, the brain samples were washed with 1xPBS once for 10 min and incubated in DAPI (1:1000, D1306, Invitrogen) diluted in 1xPBS overnight at + 4 °C on a shaker. Samples were then washed three times the next day using 1xPBS for 5 min each and mounted on slides. Finally, they were allowed to dry, cover-slipped using Epredia™Immu-Mount™ (9990402, Fisher Scientific) mounting media and stored at + 4 °C until imaging.

For immunofluorescence staining of the brain sections, samples were washed with 1xPBS containing 0.1% Triton-X-100 (PBS-T) for 10 min at RT on a shaker. The samples were then treated with Tris-EDTA (pH 8.0) at 80 °C for 30 min for antigen retrieval and cooled down at RT for 1 h before washing with PBS-T three times for 10 min each at RT. Brain sections were then incubated in blocking solution (5% normal donkey serum (Jackson ImmunoResearch), 1% bovine serum albumin and 0.5% Triton X-100 in 1xPBS) overnight on a shaker at + 4 °C. Samples were incubated in primary and secondary antibodies (Supplementary Table 2), diluted in blocking solution, for 72 h at + 4 °C each with three times 10 min washing with PBS-T in between. After three times 10 min washing with 1xPBS, the samples were mounted on objective glass and cover-slipped using Epredia™Immu-Mount™ (9990402, Fisher Scientific) mounting media. Imaging was performed within a week of staining. Similar steps to those described above, apart from the antigen retrieval step, were followed for Aβ staining of the cortex samples and brain sections (three sections per animal 600 μm apart ranging from bregma A/P -1.2 to 3.0). Methoxy (4920, Tocris) diluted in 1xPBS was added to the cortical brain samples on the final day of incubation and they were then incubated for a further 24 h on the shaker at + 4 °C followed by washing with 1xPBS and sample clearing.

### Tissue clearing

After immunostaining, the cortex samples were incubated in 50% CUBIC-1 (v/v) reagent [85] (diluted in 1xPBS) overnight at RT on a shaker. Samples were wrapped in foil to protect them from light. The following day, samples were transferred to 100% (v/v) CUBIC-1 reagent on a shaker at RT and kept protected from light. The solution was changed every second day for 10 days. The samples were imaged after 10 days of treatment and imaging finished within 1 week.

### Tissue imaging and image analysis

All the staining, imaging and analyses were performed in a blind manner. Brain sections (from drainage, efflux and AD experiments), skull whole mounts and lymph nodes (from drainage and efflux experiments) were imaged using AxioZoom V16 stereomicroscope (Carl Zeiss), equipped with an Orca-Fusion BT sCMOS camera (Hamamatsu Systems), and using Zen 3.3 pro software (Carl Zeiss). The objectives used were PlanNeoFluar Z 2.3x and PlanNeoFluar Z 1.0x and the imaging parameters were same for all the samples. Analysis of the acquired images was performed using the Fiji processing package of Image J2 software (Version 1.53, National Institute of Health). For the brain tracer analyses, the background was subtracted uniformly from the brain sections. The slices were outlined manually, auto-thresholded and their area fractions were measured. The average value of eight sections per animal was reported. To quantify the Aβ load in the brain sections, brain sections were outlined using a freehand tool from ImageJ. The images were thresholded and analyse particle plugin (circularity 0–1, size 4-infinity) was used. The average value of three sections per mouse was reported.

A free hand tool was used to make a ROI and the mean pixel intensities of the dcLNs for each mouse were measured, and the average value per mouse was reported. For analysis of LYVE1 staining from skull whole mounts, a freehand tool was used to highlight the regions manually and thresholding was utilized to measure the area fraction.

Immunofluorescence staining of the brain sections was imaged using a Leica STELLARIS 8 DIVE or Leica SP8 Falcon with a DMI8 microscope using LASX (4.5.0) acquisition software. The objectives used were HC PL APO 10x/0.40 CS2 (air), HC PL APO 20x/0.75 CS2 (air) and HC PL APO 63x/1.40 W motCORR CS2 (oil immersion) and the image acquisition settings being the same for all the samples. The images acquired were analysed using the Fiji processing package of Image J2 software (Version 1.54, National Institute of Health). Thresholding tool was used to measure the area fraction for each ROI, and the average value of ten ROIs per animal was reported. To analyse ERTR7 staining of the brain sections, a line tool from ImageJ was used to measure the length of the ERTR7 positive vessels and the length of a vessel that was positive for the CSF injected tracer, after which the exported data were further analysed using Excel. The average value of twenty ROIs per animal was reported.

The cleared brain cortical samples were imaged using Leica SP8 Falcon with a DMI8 microscope using LASX (4.5.0) acquisition software. The objective used was HC PL APO 40x/1.10 W motCORR CS2 (water immersion) and the image acquisition settings were kept constant for all the samples. The images were rendered in 3D with Imaris Software (Bitplane) for analysis and the surface was added using the same thresholding parameters. The volume of vessels from the brain cortical samples was calculated, the statistics were exported, and the average value of three ROIs per animal was reported. In addition to the surfaces of the vessels in the cortical samples, surfaces were also added to Methoxy^+^ plaques. Col IV^+^ and αSMA^+^ vessels were identified as arteries and only the plaques adjacent to the arteries were quantified. The volume of plaques was calculated, exported and an average value of three ROIs per animal was reported.

### Statistical analysis

The statistical analysis was carried out using GraphPad Prism 10 (GraphPad Software). All values are reported in the figures as mean ± standard error of the mean (SEM). The normality of the distribution of the data was tested using the Shapiro-Wilk test, and the unpaired two-tailed student’s t-test with Welch’s correction was used to compare differences between two groups in the normally distributed data and the Mann-Whitney unpaired two-tailed student’s t-test when the data were not normally distributed. One-way ANOVA followed by the Tukey post-hoc test was used for comparisons between more than two groups with one variable and two-way ANOVA followed by Sidak’s post-hoc test for comparisons between multiple groups with two or more variables. Differences were considered statistically significant at *p* < 0.05.

## Electronic supplementary material

Below is the link to the electronic supplementary material.

Supplementary Material 1

## Data Availability

No datasets were generated or analysed during the current study.

## Abbreviations

AD: Alzheimer’s disease
ANOVA: Analysis of variance
αSMA: Alpha smooth muscle actin
Aβ: Amyloid beta
CM: Cisterna magna
COS: Confluence of sinus
CSF: Cerebrospinal fluid
dcLN: Deep cervical lymph node
FITC: Fluorescein isothiocyanate
FRAP: Fluorescent recovery after photobleaching
GAG: Glycosaminoglycan
HSPG: Heparan sulphate proteoglycan
ISF: Interstitial fluid
LYVE1: Lymphatic vessel endothelial hyaluronan 1
PVS: Perivascular space
SEM: Standard error of mean
