# Supplementary material for: The glycosaminoglycan chains of perlecan regulate the perivascular fluid transport
Source: Fluids Barriers CNS. 2025 May 8;22:48. doi: 10.1186/s12987-025-00648-7 (PMC12063283; doi:10.1186/s12987-025-00648-7)
Supplement: Supplementary file 1 — Supplementary Material 1 [file 12987_2025_648_MOESM1_ESM.docx]

*
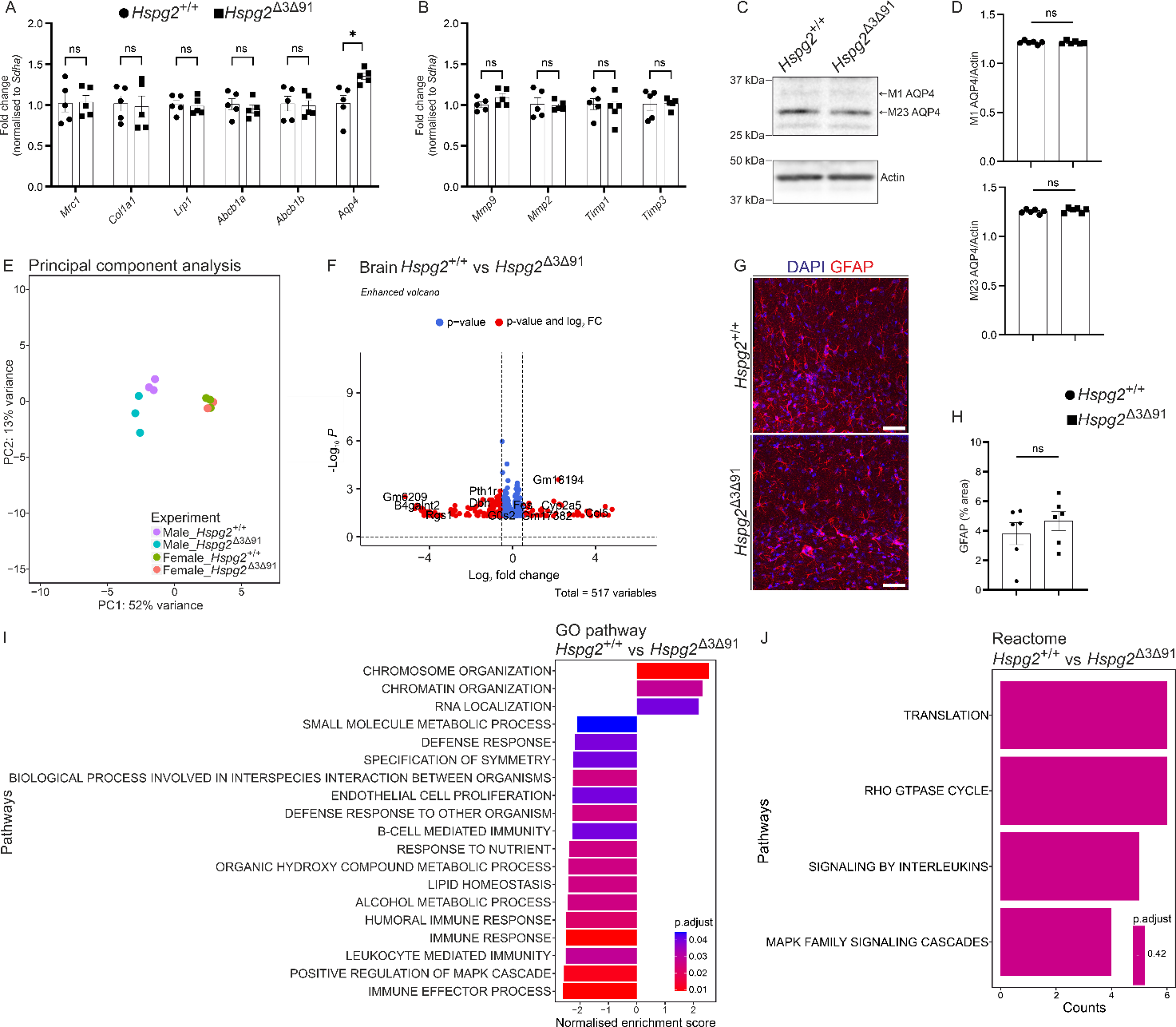
*

#### ***Supplementary Figure 1*** *-* ***Gene and protein expression profiles in the* Hspg2^∆3∆91^ *brains****. A-B) qPCR analysis of markers of perivascular cells (Mrc1, Col1a1), protein transporters (Lrp1, P-glycoprotein, Abcb1a, Abcb1b) and matrix remodelling (Mmp2/9, Timp1/3). Note a statistically significant increase in Aqp4 in the* Hspg2^∆3∆91^ *compared to Hspg2^+/+^ mice. n=5 mice per genotype. C-D) Representative image and quantification of western blotting of aquaporin 4 (AQP4) protein. n=6 mice per genotype. Two bands in the 31-35 kDa range were detected as previously reported MWs* [1] *of the m1 and m23 isoforms of AQP4. E) Principal component analysis of brain samples from the Hspg2^+/+^ (n= 6) and* Hspg2^∆3∆91^ *(n=5) mice. F) Volcano plot of differentially expressed genes between Hspg2^+/+^ and* Hspg2^∆3∆91^*. G-H) Representative images and quantification of brain sections from the Hspg2^+/+^ and* Hspg2^∆3∆91^ *mice stained with DAPI (blue) and glial fibrillary acidic protein (GFAP, red), n=6 mice per genotype. I) Pathway enrichment analysis of differentially expressed genes from Hspg2^+/+^ and* Hspg2^∆3∆91^*. Colour of the bar corresponds to the adjusted p-value of the enriched pathways, while the length corresponds to the normalized enrichment score. J) Pathway enrichment analysis of differentially expressed proteins from the proteomic analysis of the brain hemispheres. The proteins were mapped to pathways from the reactome database. Box length represents the number (count) of proteins in each pathway and colour corresponds to the adjusted p-value. Scale bar, 50 µm (G). The statistical tests used were the multiple unpaired two-tailed t-test with Welch’s correction followed by Benjamini and Hochberg correction for multiple tests (A, B), the Mann-Whitney unpaired two-tailed t-test (D) and the unpaired two-tailed t-test with* Welch’s correction (H). *p < 0.05. ns, non-significant. Mean ± SEM.


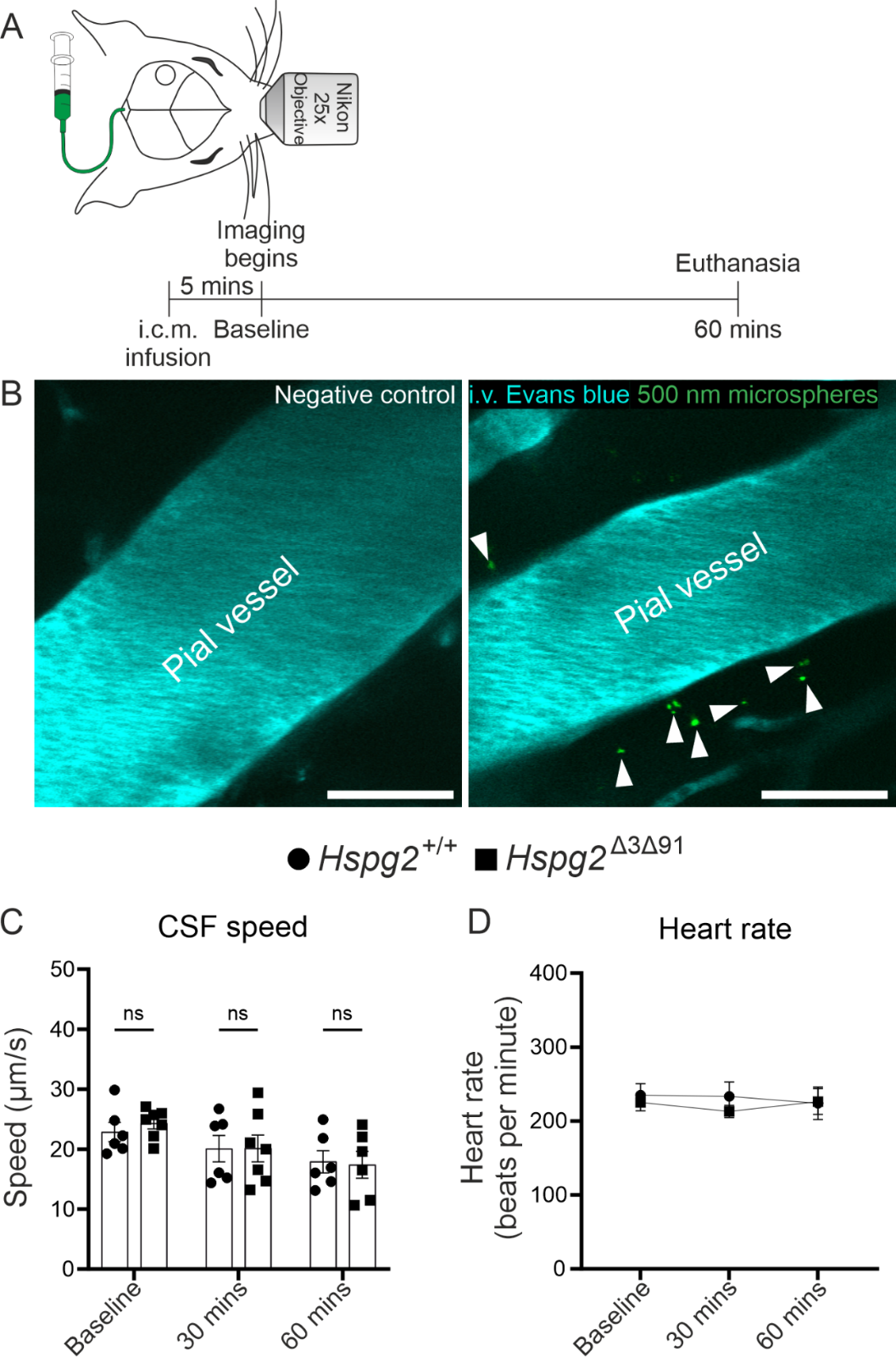


#### ***Supplementary Figure* 2** – **Cerebrospinal fluid tracer velocity in the perivascular space.** A) Schematic timeline of the imaging experiment with the location of the cranial window (circle). B) Illustrative images of pial vessels (i.v. Evans blue, pseudo-coloured in turquoise) with 500 nm microspheres in the perivascular spaces (green, white arrowheads). Note that the negative control (left) shows only the pial vessel. C) Quantification of bead movement speeds in the Hspg2^+/+^ (n=6) and Hspg2^∆3∆91^ (n=7) mice at the time points indicated. D) Heart rate measurements show no significant differences between the genotypes throughout the experimental timeline. Scale bar, 20 µm (B). The statistical test used was 2-way ANOVA with repeated measures followed by Sidak’s post-hoc test (C, D). ns, non-significant. Mean ± SEM.


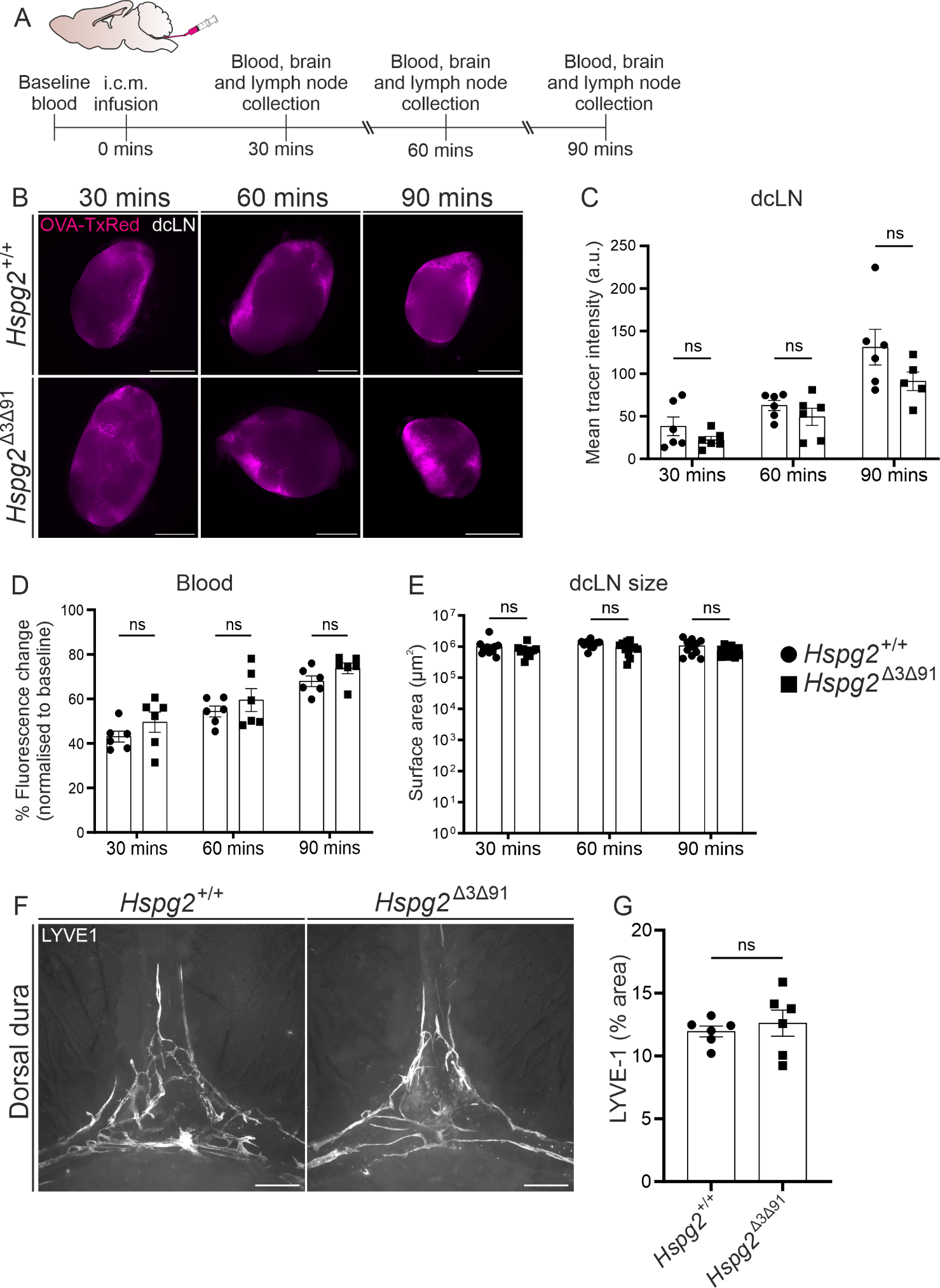


#### ***Supplementary Figure* 3** - **Drainage of cerebrospinal fluid into the deep cervical lymph nodes and blood**. A) Schematic timeline of the experiment and sample collection. B) Images of deep cervical lymph nodes (dcLNs) from Hspg2^+/+^ and Hspg2^∆3∆91^mice at the time points indicated, showing the presence of fluorescent tracer (magenta). C) Quantification of fluorescence intensity in the dcLNs at the time points indicated. D) Serum fluorescence analyses at the time points indicated. E) Quantification of the surface areas of dcLNs from Hspg2^+/+^ and Hspg2^∆3∆91^mice. C-E) 30 mins (n=6), 60 mins (n=6), 90 mins (n=5-6 mice per genotype). F) Lymphatic vessels in the meningeal dura of the Hspg2^+/+^ and Hspg2^∆3∆91^ mice. G) Quantified image data for F (n=6 mice per genotype). Scale bar, 500 µm (B), 1 mm (F). The statistical tests used were 2-way ANOVA followed by Sidak’s post-hoc test (C-E) and the unpaired two-tailed t-test with Welch’s correction (G). ns, non-significant. Mean ± SEM.


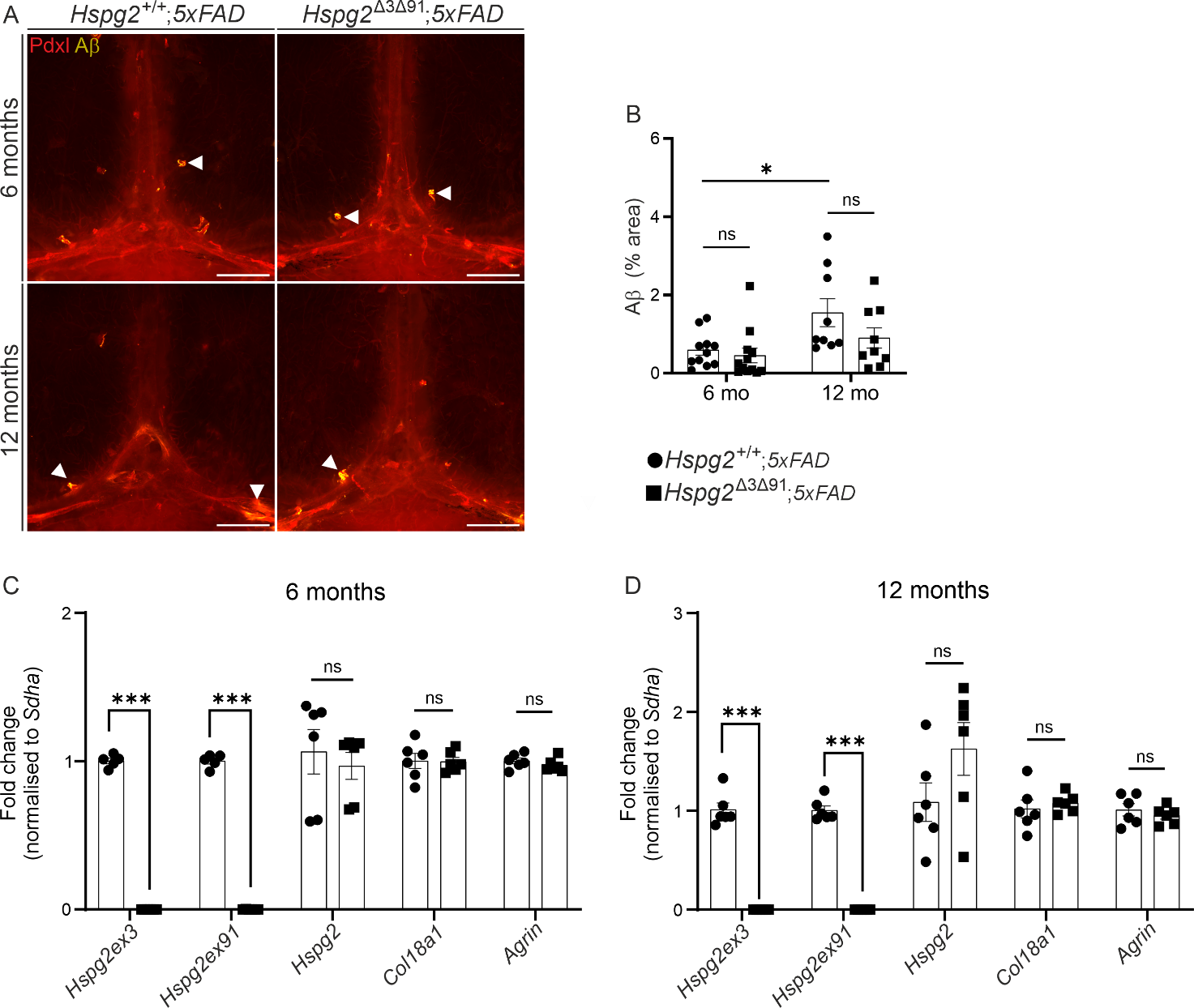


#### ***Supplementary Figure* 4 - Accumulation of amyloid-β (Aβ) in the meningeal dura and expression of basement membrane (BM) components in the Hspg2^∆3∆91^;5xFAD mice**. A) Dural samples stained with podocalyxin (PDXL, red) and Aβ (yellow) antibodies from the Hspg2^+/+^;5xFAD and Hspg2^∆3∆91^;5xFAD mice. B) Quantification of the Aβ immunoreactive area at age 6 months (n=12 mice per genotype) and 12 months (n=9 mice per genotype). C, D) qPCR analysis of brain hemispheres from the Hspg2^∆3∆91^;5xFAD mice shows no statistically significant changes in the expression level of BM heparan sulphate proteoglycans such as perlecan (Hspg2), Col18a1 and agrin relative to Hspg2^+/+^;5xFAD at age 6 months (n=6 mice per genotype) or 12 months (n=6 mice per genotype). Analysis of the deleted exons 3 and 91 showed no qPCR amplicon in the Hspg2^∆3∆91^;5xFAD mice relative to the Hspg2^+/+^;5xFAD mice. Scale bar, 1 mm (A). The statistical tests used were 2-way ANOVA with Sidak’s post-hoc test (B) and the multiple unpaired two-tailed t-test with Welch’s correction followed by Benjamini and Hochberg correction for multiple tests (C, D). *p < 0.05, ***p < 0.05. ns, non-significant. Mean ± SEM.

| **Primer** |  | **Sequence** | **Application** |
| --- | --- | --- | --- |
| *Hspg2ex2* | Forward | *GTGACGCATGGGCTGAGGGCCTACGATG* | qPCR |
|  | Reverse | *TCTTCCTGTATTCGGGCTCCAG* |  |
| *Hspg2ex3* | Forward | *CGGTATGGCTGGACCTACTC* | qPCR |
|  | Reverse | *GCCCGGAAATAGACCATCTG* |  |
| *Hspg2ex4* | Forward | *GAGAGGTGTCGGAGGCTGTGG* | qPCR |
|  | Reverse | *CAGGCGTCGGAACTTGAACCC* |  |
| *Hspg2ex91* | Forward | *GCGCTGGATATGGGGTAGTG* | qPCR |
|  | Reverse | *ATTGCCCAGGGGCATCATTG* |  |
| *Hspg2ex94* | Forward | *GGCATCGGATAACAGCGCTGCG* | qPCR |
|  | Reverse | *CCTGCCTGGGCGCGATGCTGC* |  |
| *Ex3-wt* | Forward | *TCCTGAACGCCTAGCCTCATGATCCCG* | Genotyping |
|  | Reverse | *GCCCACGTCTCCGCTGCCGAGGCCATC* |  |
| *Ex3-mut* | Forward | *TCCTGAACGCCTAGCCTCATGATCCCG* | Genotyping |
|  | Reverse | *CAGACTGCCTTGGGAAAAGCGCCT* |  |
| *Ex91-wt* | Forward | *GCGGGCGAATATGAGTTCCAGTGC* | Genotyping |
|  | Reverse | *GCCTTCAGGGTGCCAGTCTGACTCCAC* |  |
| *Ex91-mut* | Forward | *GCGGGCGAATATGAGTTCCAGTGC* | Genotyping |
|  | Reverse | *ACCCACATGGCTGTACAGCTCCCTATA* |  |
| *Neo* | Forward | *TGTCATCTCACCTTGCTCCTG* | Genotyping |
|  | Reverse | *TCAAGAAGGCGATAGAAGGCG* |  |
| *Col18a1* | Forward | *CCAGGACAGCAGGGTGTG* | qPCR |
|  | Reverse | *CCTGGTGGCCCAGGTG* |  |
| *Aqp4* | Forward | *ATCTTTCCACCCCTACTCTCCA* | qPCR |
|  | Reverse | *AGGAATGTCCACACTTACCCCAC* |  |
| *Agrin* | Forward | *CTAGGGGAATCTCCGGTCCC* | qPCR |
|  | Reverse | *GGCCTCTTAGAGACACCAGC* |  |
| *Mrc1* | Forward | *TTCAGCTATTGGACGCGAGG* | qPCR |
|  | Reverse | *GAATCTGACACCCAGCGGAA* |  |
| *Lama2* | Forward | *TGAGTATGAAAGCAAGGCCAGA* | qPCR |
|  | Reverse | *CAATAGACACAGCGGCAAGC* |  |
| *Lama4* | Forward | *AGTGTCGCAAGCAAGATCCA* | qPCR |
|  | Reverse | *TGCTTCCGAGGTAGAGGACA* |  |
| *Abcb1a* | Forward | *TTCTCTTTGTCCGCGGAGTC* | qPCR |
|  | Reverse | *CTGTGCCTGGGCTTTCTTGA* |  |
| *Abcb1b* | Forward | *CAGTGGCTCTTGAAGCCGTA* | qPCR |
|  | Reverse | *AACTCCATCACCACCTCACG* |  |
| *Lrp1* | Forward | *GCGGTGTGACAACGACAATG* | qPCR |
|  | Reverse | *GGTCTTGTAGCCTGGTTGGT* |  |
| *Lama5* | Forward | *CCAGCAAGGCGATCCAAGT* | qPCR |
|  | Reverse | *TCCTGGTCTACGCTGAACAC* |  |
| *Sdha* | Forward | *GCTCCTGCCTCTGTGGTTGA* | qPCR |
|  | Reverse | *AGCAACACCGATGAGCCTG* |  |
| *Timp1* | Forward | *CCCCAGAAATCAACGAGACCA* | qPCR |
|  | Reverse | *ACTCTTCACTGCGGTTCTGG* |  |
| *Timp3* | Forward | *CTTTGTGGAGAGGTGGGACC* | qPCR |
|  | Reverse | *ATGCAGGCGTAGTGTTTGGA* |  |
| *Mmp2* | Forward | *AGGAGCTCTATGGGCCCTC* | qPCR |
|  | Reverse | *GCAGATCTCCGGAGTGACAG* |  |
| *Mmp9* | Forward | *CGACATCTTCCAGTACCAAGACAA* | qPCR |
|  | Reverse | *TTGGAAACTCACACGCCAGA* |  |
| *Col1a1* | Forward | *ATCAGCTGGAGTTTCCGTGC* | qPCR |
|  | Reverse | *GGACCCATTGGACCTGAACC* |  |

#### **Supplementary Table 1**- List of primers used for genotyping and qPCR.

| **Antibody** | **Origin/**  **reactivity** | **Dilution** | **Usage** | **Catalogue number** |
| --- | --- | --- | --- | --- |
| AQP-4 | Rabbit anti-mouse | 1:500 | Western blot | AB3594, Merck |
| Perlecan | Rabbit anti-mouse | 1:2000 | Western blot | Gift from Takako Sasaki [2] |
| Actin | Rabbit anti-mouse | 1:1000 | Western blot | A2066, Sigma |
| Vinculin | Mouse anti-mouse | 1:1000 | Western blot | V4505, Sigma |
| GFAP | Rabbit anti-mouse | 1:500 | Immunofluorescence | ab7260, Abcam |
| LYVE-1 | Rat anti-mouse | 1:300 | Immunofluorescence | MAB2125, R&D Systems |
| α-SMA-Cy3 conjugated | Mouse anti-mouse | 1:300 | Immunofluorescence | C6198, Sigma |
| Collagen IV | Rabbit anti-mouse | 1:300 | Immunofluorescence | ab6586, Abcam |
| Perlecan | Rat anti-human | 1:300 | Immunofluorescence | MAB1948P, Merck |
| Podocalyxin | Goat anti-mouse | 1:500 | Immunofluorescence | AF1556, R&D Systems |
| Amyloid beta | Rabbit anti-human | 1:500 | Immunofluorescence | D54D2, Cell Signalling |
| HRP conjugated IgG | Goat anti-rabbit | 1:10000 | Western blot | 111-035-003, Jackson ImmunoResearch |
| HRP conjugated IgG | Goat anti-mouse | 1:10000 | Western blot | 115-035-003, Jackson ImmunoResearch |
| Alexa Fluor 488 | Donkey anti-rat | 1:500 | Immunofluorescence | 712-585-150, Jackson ImmunoResearch |
| Alexa Fluor 488 | Donkey anti-rabbit | 1:500 | Immunofluorescence | 711-545-152, Jackson ImmunoResearch |
| Alexa Fluor 488 | Donkey anti-goat | 1:500 | Immunofluorescence | 705-545-147, Jackson ImmunoResearch |
| Alexa Fluor 647 | Donkey anti-rat | 1:500 | Immunofluorescence | 712-605-153, Jackson ImmunoResearch |
| Alexa Fluor 647 | Donkey anti-rabbit | 1:500 | Immunofluorescence | 711-605-152, Jackson ImmunoResearch |

#### ***Supplementary Table 2*** - List of antibodies used.

**References –**

1. Furman CS, Gorelick-Feldman DA, Davidson KG V., Yasumura T, Neely JD, Agre P, et al. Aquaporin-4 square array assembly: Opposing actions of M1 and M23 isoforms. Proc Natl Acad Sci [Internet]. 2003;100:13609–14. Available from: https://pnas.org/doi/full/10.1073/pnas.2235843100

2. Brown JC, Sasaki T, Göhring W, Yamada Y, Timpl R. The C‐Terminal Domain V of Perlecan Promotes β1 Integrin‐Mediated Cell Adhesion, Binds Heparin, Nidogen and Fibulin‐2 and Can be Modified by Glycosaminoglycans. Eur J Biochem [Internet]. 1997;250:39–46. Available from: https://febs.onlinelibrary.wiley.com/doi/10.1111/j.1432-1033.1997.t01-1-00039.x
